# Antibiotics for amniotic-fluid colonization by *Ureaplasma* and/or *Mycoplasma* spp. to prevent preterm birth: A randomized trial

**DOI:** 10.1371/journal.pone.0206290

**Published:** 2018-11-07

**Authors:** Gilles Kayem, Alexandra Doloy, Thomas Schmitz, Yvon Chitrit, Philippe Bouhanna, Bruno Carbonne, Jean Marie Jouannic, Laurent Mandelbrot, Alexandra Benachi, Elie Azria, Francoise Maillard, Florence Fenollar, Claire Poyart, Cécile Bebear, François Goffinet

**Affiliations:** 1 Inserm UMR 1153, Obstetrical, Perinatal and Pediatric Epidemiology Research Team (Epopé), Center for Epidemiology and Statistics Sorbonne Paris Cité, DHU Risks in pregnancy, Paris Descartes University, Paris, France; 2 Sorbonne University, Université Pierre et Marie Curie, Paris, France; 3 Department of Obstetrics and Gynecology, CHI Créteil Hospital, Créteil, France; 4 Department of Microbiology, Cochin, Broca, Hôtel Dieu Hospital, AP-HP, Paris, France; 5 Paris Diderot-Paris VII University, Paris, France; 6 Department of Gynecology and Obstetrics, Robert Debré Hospital, APHP, Paris, France; 7 Department of Gynecology and Obstetrics, Louis Mourier Hospital, APHP, Paris, France; 8 Department of Gynecology and Obstetrics, CHI Poissy, Poissy, France; 9 Department of Obstetrics and Gynecology, Saint Antoine Hospital, APHP, Paris, France; 10 Department of Obstetrics and Gynecology, Trousseau Hospital, APHP, Paris, France; 11 Inserm IAME-UMR1137, Paris, France; 12 Department of Obstetrics and Gynecology, Antoine Beclère Hospital, Clamart, APHP, Paris, France; 13 Department of Gynecology and Obstetrics, Bichat Hospital, APHP, Paris, France; 14 Unité de Recherche sur les Maladies Infectieuses et Tropicales Emergentes CNRS UMR 6236 IRD 198, Marseille, France; 15 Paris Descartes University, Paris, France; 16 INRA, USC EA 3671, Mycoplasmal and Chlamydial Infections in Humans, Bordeaux, France; 17 University of Bordeaux, USC EA 3671, Mycoplasmal and Chlamydial Infections in Humans, Bordeaux, France; 18 Department of Obstetrics and Gynecology, Cochin, Broca, Hôtel Dieu Hospital, AP-HP, Paris, France; University of Western Australia, AUSTRALIA

## Abstract

**Objective:**

To assess whether antibiotics used for treatment in asymptomatic second-trimester women positive for *Mycoplasma* or *Ureaplasma* spp. detected by amniotic-fluid PCR prevents preterm delivery.

**Design:**

A randomized, double-blind, placebo-controlled trial.

**Setting:**

10 maternal fetal medicine centers in France.

**Population:**

Women with a singleton pregnancy who underwent amniocentesis between 16 and 20 weeks’ gestation (weeks) for Down syndrome screening. A sample of 238 women with PCR-positive findings per treatment group was needed to show a 50% reduction in the preterm delivery rate.

**Methods:**

Amniotic fluid was tested. Women with positive findings on real-time PCR of amniotic fluid for *Mycoplasma hominis*, *Mycoplasma genitalium*, *Ureaplasma urealyticum* and *Ureaplasma parvum* were randomized to receive josamycin or placebo. Amniotic fluid was also tested for 16S PCR.

**Main outcome measures:**

The primary outcome was delivery before 37 weeks.

**Results:**

In total, 1043 women underwent amniotic-fluid screening with specific PCR detection between July 2008 and July 2011: PCR detection failed in 27 (2.6%), and 20 (1.9%) underwent termination of pregnancy. Among the 1016 women with PCR results, 980 had available data for the primary outcome (delivery before 37 weeks) and 29 (3.0%) were positive for *Ureaplasma* and/or *Mycoplasma* spp. Because of the low rate of women with PCR-positive findings, the trial was stopped prematurely. In total, 19 women were randomized to receive placebo (n = 8) or josamycin (n = 11) and their characteristics were comparable, as was the rate of preterm delivery and secondary outcomes. In comparing all PCR-positive and -negative women regardless of treatment, PCR positivity for *Ureaplasma* and/or *Mycoplasma* spp. was not associated with any adverse pregnancy or neonatal outcome. Amniotic-fluid screening by 16S PCR showed no other bacterial colonization associated with preterm birth.

**Conclusions:**

Because of a low amniotic fluid colonization rate, the trial was interrupted. Maternal amniotic-fluid colonization by *Mycoplasma* and/or *Ureaplasma* spp. at 16–20 weeks in asymptomatic women is rare and not associated with adverse pregnancy outcomes.

**Trial registration:**

ClinicalTrials.gov NCT00718705

## Introduction

Preterm delivery represents 5.5% to 12% of deliveries in high-income countries and is a major cause of infant morbidity and mortality [1]. Neonatal mortality occurs in 60% of cases in children born before 30 weeks’ gestation (weeks), and half the neurological, cognitive and respiratory effects are observed before 32 weeks [2]. Infection could account for up to 50% of spontaneous preterm deliveries [3].

In women with spontaneous preterm labor and intact membranes, the most commonly identified bacteria in amniotic fluid are *Ureaplasma* spp., *Mycoplasma hominis*, *Gardnerella vaginalis*, peptostreptococci, and *Bacteroides* species—all vaginal organisms of relatively low virulence and all associated with increased rate of preterm delivery and chorioamnionitis [4]. The leading hypothesis is that these vaginal organisms may ascend first into the choriodecidual space early in pregnancy, remain undetected for months and finally result in the production of pro-inflammatory cytokines, chemokines and prostaglandins that cause cervical ripening, uterine contractions, rupture of membranes and preterm delivery [3]. Mycoplasmas and ureaplasmas have been detected in 6% and 11% of cases by PCR in amniotic fluid obtained for routine chromosomal analysis during the second trimester in asymptomatic women. Their presence in amniotic fluid was found associated with preterm delivery [5, 6, 7].

Etiologic treatments to prevent preterm birth are lacking, and the potential benefit of the treatment for *Mycoplasma* or *Ureaplasma* infection in second-trimester amniotic fluid has never been evaluated [8]. We performed a randomized, prospective, multicenter, double-blind, placebo-controlled trial to assess the effect of antibiotics on reducing the risk of preterm delivery in second-trimester asymptomatic women who were PCR-positive for mycoplasmas, including *Ureaplasma* spp. (*U*. *parvum* and *U*. *urealyticum*) and *M*. *hominis* and *M*. *genitalium*. Josamycin was the antibiotic used because at the time of the study, it was the sole antibiotic authorized for pregnant women in France and active against the studied *Mycoplasma* and *Ureplasma* spp. [9].

## Materials and methods

The trial was approved by the national data protection authority (Commission Nationale de l’Informatique et des Libertés, CNIL) and by the committee for the protection of people participating in biomedical research (Comité de Protection des Personnes [CPP], CPP Poissy Saint Germain, no. 07051). This trial was registered at ClinicalTrials.gov (no. NCT00718705).

### Participants

Women were recruited between July 2008 and July 2011 from 10 hospitals in the Paris region that had a prenatal diagnosis center. Women with a singleton pregnancy who underwent amniocentesis from 16 to 20 weeks for Down syndrome screening were eligible, and amniotic fluid was tested for *U*. *urealyticum*, *U*. *parvum*, *M*. *hominis and M*. *genitalium* by PCR. Exclusion criteria were age < 18 years, known allergy to macrolides or lactose (included in placebo), known major structural or chromosomal fetal abnormality, and not speaking or understanding French. All participants gave written informed consent at 2 stages: before amniocentesis to agree to PCR analysis and, for those with PCR-positive results, before randomization.

### Intervention

Women PCR-positive for *Mycoplasma* or *Ureaplasma* spp. were randomized to receive josamycin (Bayer) (1 g/day for 10 days) or placebo tablets. Each participant was given 20 tablets in 2 blister packs of 10 tablets each in one sealed box.

Josamycin was used because at the time of the study, it was the sole antibiotic authorized for pregnant women in France and it is active against the studied *Mycoplasma* and *Ureplasma* spp. *Ureaplasma* spp. are susceptible to macrolides but not lincosamides [9]. Conversely, *M*. *hominis* is resistant to 14- and 15-member macrolides such as erythromycin but is sensitive to 16-member macrolides such as josamycin and lincosamides. *M*. *genitalium* is intrinsically susceptible to all macrolides; however, its increasing resistance rate to azithromycin is a concern [10]. Josamycin shows minimal inhibitory concentrations of <1 and <2 mg/l for *M*. *hominis* and *Ureaplasma* spp., respectively [11].

### Randomization and masking

Staff at the Clinical Research Unit, Carre Saint Louis, Paris, France, created a randomization sequence with a 1:1 ratio. Boxes of josamycin and placebo were packed and labeled by the National Agency of Drug Safety (Paris) according to this randomization sequence and shipped to each participant hospital. The randomization sequence, consisting of batches of 18 boxes with permuted blocks of randomly mixed sizes (2, 4 or 6) stratified by center, was created by using an interactive internet randomization system. All participants were blinded to treatment assignment for the duration of the trial, and the randomization code was not broken before all data had been collected, including the pregnancy and neonate follow-up until hospital discharge. Only the statistician and the independent Data Monitoring and Safety Committee had access to unblinded data during the study period. None of these people had any contact with participants in the study.

### Procedure

Women were included between 15+0 and 20+6 weeks when amniocentesis was performed. An amount of 1 ml amniotic fluid was sent for real-time PCR in the microbiological center unit of Cochin hospital. If the PCR test was positive, a medical visit was scheduled and women were asked if they would agree to randomized treatment.

Gestational age was based on crown–rump length measured during the first trimester sonography. Treatment started between 20+0 and 23+6 weeks and was self-administered daily by participants for 10 days.

Demographic and obstetric characteristics were collected. Participants were interviewed by telephone after the end of the treatment to assess side effects and compliance.

### Real-time PCR for *Ureaplasma* and *Mycoplasma* spp.

One ml of amniotic fluid, to which 10 μL of viral DNA used as control (Dia-IC/DNA(YD)-050, now renamed DICD-YD-L100, Diagenode), was extracted by using the NucliSENS easyMAG system (bioMérieux, France) according to the manufacturer’s instructions. Three distinct real-time PCR tests (U. urealyticum and U. parvum, M. genitalium, and M. hominis) were performed with LightCycler 2.0 (Roche). A sample was considered positive when any of the three tests reached a positive signal, given that the internal control (viral DNA) was positive.

*U*. *urealyticum* and *U*. *parvum* real-time PCR (targeting 90 bp of the *urease* gene for *U*. *urealyticum* [12]) involved the primers UU-1524R (5’-TTCCTGTTGCCCCTCAGTCT-3’) and UU-1623F (5’-AAGGTCAAGGTATGGAAGATCCAA-3’) and Taqman probes UU-parvo (5’-FAM-TCCACAAGCTCCAGCAGCAATTTG-TAMRA-3’) and UU-T960 (5’-Yakima Yellow-ACCACAAGCACCTGCTACGATTTGTTC-TAMRA-3’). *M*. *genitalium* PCR targeting 78 bp of the *MgPa* adhesin gene involved the primers MG-Pa-432R (5’-GTTAATATCATATAAAGCTCTACCGTTGTTATC-3’) and MG-Pa-355F (5’-GAGAAAT-ACCTTGATGGTCAGCAA-3’), and the Taqman probe MG-Pa380 (5’-FAM-ACTTTGCAATCAGAAGGT-NFQMGB-3’).[13] *M*. *hominis* real-time PCR targeting 94 bp of the *yidC* gene involved the primers MHyidCfwd (5’-TCACTAAACCGGGTATTTTCTAACAA-3’) and MHyidCrev (5’- TTGGCATATATTGCGATAGTGCTT-3’) and the Taqman probe MHyidC (5’-FAM- CTACCAATAATTTTAATATCTGTCGGTATG-BHQ-3’) [14]. A 20-μl PCR reaction mixture containing 5 μM each primer (final concentration 0.5 μM), 2.5 μM each probe (final concentration 0.25 μM), LightCycler FastStart DNA Master Hybprobe (Roche), and 5 μl sample extract was amplified for 10 min at 95°C, followed by 50 cycles of 15 s at 95°C and 1 min at 60°C.

### 16S rDNA PCR detection

Molecular analyses with broad-spectrum PCR and sequencing were performed to target 16S rDNA, as described [15, 16]. All primers used for PCR and sequencing of 16S rDNA are in S1 Table [15, 16]. PCR products were purified by using the PCR kit Nucleofast 96 (Macherey-Nagel, Hoerdt, France), and sequencing involved use of the Big Dye Terminator v1.1 sequencing kit (Applied Biosystems, Foster City, CA, USA). Products of the sequencing reaction were purified, and sequences were analyzed on an ABI PRISM 3130X Genetic Analyzer (Applied Biosystems, Foster City, CA, USA) [15]. The sequences were assembled and amended by using CodonCode Aligner v4.1.1 (CodonCode Corp., USA). Then, a correct consensus sequence was saved and compared with the GenBank database by using BLAST software (http://blast.ncbi.nlm.nih.gov/Blast.cgi). An isolate was correctly identified when it yielded > 98.7% sequence identity for the 16S rRNA sequence with the closest bacterial species sequence in GenBank [15–17].

### Outcomes

The primary outcome was delivery before 37 weeks. Pre-specified secondary outcomes were 1) delivery before 22, 28 and 32 weeks; 2) number of liveborn infants; 3) hospital admission for preterm labor or preterm premature rupture of membranes (PPROM); 4) birth weight; and 5) selected neonatal complications including neonatal mortality, neonatal infection, respiratory distress syndrome, oxygen requirement at 36 weeks, intraventricular hemorrhage grade 1–4 according to Papile criteria [18], and periventricular leucomalacia and necrotizing enterocolitis according to the Bell classification [19]. Early-onset neonatal infection was defined by positive bacteriology in blood or cerebrospinal fluid.

### Statistical analysis

A sample size of 3200 was needed to detect 476 women (15%) who were PCR-positive for *Ureaplasma* or *Mycoplasma* spp., 238 women per treatment group, to show a 50% reduction in the preterm delivery rate from 17% to 8.5% with use of antibiotics, with a two-sided test (α error 5%, power 80%). The PCR-positive rate and 17% preterm delivery rate for PCR-positive women were estimated according to what was previously reported for second-trimester asymptomatic women at the time of the conception of the study [5, 6, 7].

All analyses were performed according to the intent-to-treat principle. All tests of significance were two-tailed and p < 0.05 was considered statistically significant. Comparisons between quantitative variables involved Kruskall Wallis test. For categorical variables, we used chi-square or Fisher exact test, as appropriate. All data management and analysis involved use of Stata 13.0 (Statacorp, College Station, TX, USA).

## Results

**Fig 1:**
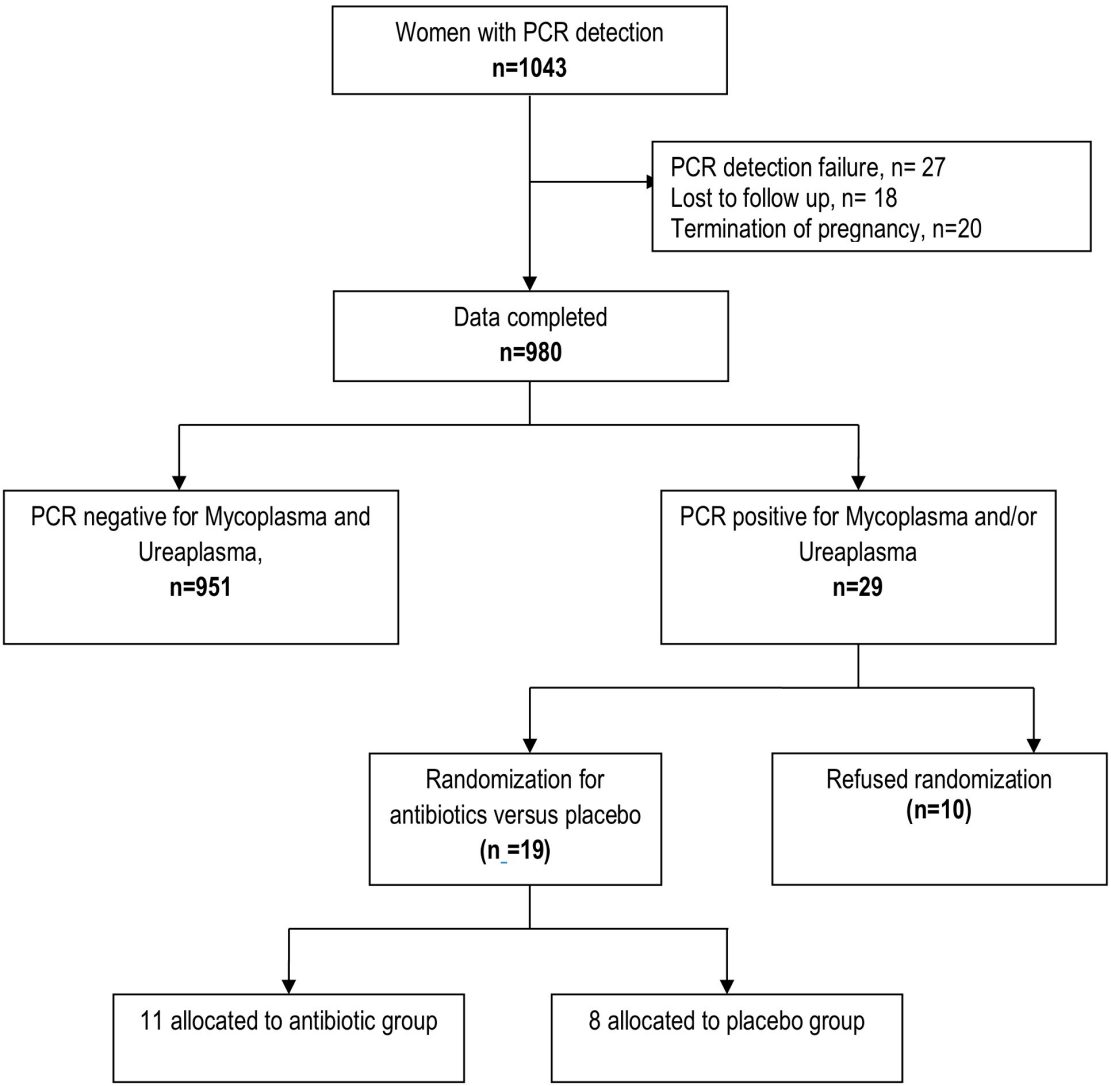
Flow chart.

Over 3 years, 1043 women underwent amniotic-fluid screening for Down syndrome and specific PCR detection; for 27 (2.6%), PCR detection failed, and 20 (1.9%) underwent termination of pregnancy for medical reasons (2 were PCR-positive). In total, 18 women (1.7%) were lost to follow-up (all PCR-negative, except 2 with PCR detection failure) (Fig 1). The global rate of PCR positivity was 31/1016 (3%, 95% confidence interval 2–4%). Among the 1016 women with PCR results, 980 had available data for the primary outcome (delivery before 37 weeks) and 29 (3.0%) were positive for *Ureaplasma* and/or *Mycoplasma* spp.: 13 (1.3%) for *M*. *genitalium*, 10 (1%) for *U*. *urealyticum*, 7 (0.7%) for *M*. *hominis* and 2 (0.2%) for *U*. *parvum*. One woman was positive for *U*. *urealyticum*, *M*. *hominis* and *M*. *genitalium* and one for *U*. *urealyticum* and *U*. *parvum*.

In all, 19 women agreed to randomized treatment with placebo (n = 8) or josamycin (n = 11). Characteristics of the 2 groups were comparable (Table 1) and the rate of preterm delivery and secondary outcomes was also similar (Table 2).

**Table 1:** Characteristics of women with PCR positivity for *Ureaplasma* and/or *Mycoplasma* spp. by treatment.

Factors studied	Josamycin n = 11	Placebo n = 8	p
Maternal age, median (IQR)	38.0 (34.0–40.0)	36.5 (34.5–39.0)	0.59
BMI, median (IQR)	21.1 (20.7–27.3)	21.2 (20.1–25.3)	0.56
Geographic origin, n (%)			
France	4 (36.4)	4 (57.1)	0.44
North Africa	4 (36.4)	1 (14.3)	
Sub-Saharan Africa	0	1 (14.3)	
Others	3 (27.3)	1 (14.3)	
Single, n (%)	0	0	-
Unemployed, n (%)	2 (18.2)	1 (14.3)	1.0
Smoker, n (%)	1 (9.1)	0	1.0
Nulliparous	8 (72.7)	6 (75.0)	1.0
Pre-existing diabetes, n (%)	0	0	-
Pre-existing hypertension, n (%)	0	0	-
Previous preterm delivery, n (%)	2 (18.2)	1 (12.5)	1.0
Previous fetal loss, n (%)	0	0	-
Previous curetage, n (%)	4 (44.4)	3 (42.9)	1.0

Percentages are of the total available data; Comparisons between quantitative variables Kruskall Wallis test. For categorical variables, chi-square or Fisher exact test was used as appropriate; BMI, body mass index; IQR, interquartile range

**Table 2:** Pregnancy outcomes for women with PCR positivity for *Ureaplasma* and/or *Mycoplasma* spp. by treatment.

Factors studied	Josamycin n = 11	Placebo n = 8	p
**Obstetrical outcomes, n (%)**			-
Preterm delivery before 37 weeks	0	0	-
Late miscarriage between 16 and 21 weeks	0	0	-
Gestational hypertension	0	0	-
Preeclampsia	0	0	-
Intra-utero fetal death	0	0	1.0
Admission for preterm labor	1 (9.1)	0	
Mode of delivery			0.21
Vaginal	6 (54.5)	4 (50.0)	
Caesarean during labor	2 (18.2)	4 (50.0)	
Planned caesarean	3 (27.3)	0	
**Neonatal outcomes**			0.56
Birth weight, median (IQR)	3480 (3140–3725)	3475 (3040–3642)	-
Neonatal infection, n (%)	0	0	1.0
Small for gestational age, n (%)	1 (9.1)	1 (12.5)	1.0
Respiratory distress syndrome, n (%)	0	1 (12.5)	-
Bronchopulmonary dysplasia, n (%)	0	0	-
Intraventricular haemorrhage, n (%)	0	0	-
Perinatal death, n (%)	0	0	

IQR, interquartile range; Comparisons between quantitative variables involved Kruskall Wallis test. For categorical variables, chi-square or Fisher exact test was used as appropriate.

In comparing all PCR-positive and -negative women, PCR positivity was not associated with any adverse pregnancy or neonatal outcome (Table 3). Among women PCR negative with previous spontaneous preterm birth, 6/33 (18%) had again a spontaneous preterm birth. Among the two women PCR positive with spontaneous previous preterm birth, one was treated by a placebo and the other one treated by josamycin. None had a preterm birth.

**Table 3:** Maternal characteristics and pregnancy outcomes by PCR positivity or negativity for *Ureaplasma* or *Mycoplasma* spp.

Factors studied	PCR-negative (n = 951)	PCR-positive (n = 29)	p
**Maternal characteristics**			
Maternal age, median (IQR)	37 (33–40)	37.0 (34–39)	0.69
BMI, median (IQR)	21.8 (20.1–24.8)	21.8 (20.1–25.3)	0.73
Geographic origin, n (%)			
France	471 (53.0)	15 (55.6)	0.67
North Africa	144 (16.2)	6 (22.2)	
Sub-Saharan Africa	65 (7.3)	2 (7.4)	
Other	208 (23.4)	4 (14.8)	
Single, n (%)	48 (5.2)	2 (7.7)	0.64
Unemployed, n (%)	148 (16.0)	6 (21.4)	0.43
Smoker, n (%)	115 (12.4)	3 (10.3)	0.74
Nulliparous, n (%)	306 (32.5)	8 (27.6)	0.69
Previous preterm delivery, n (%)	55 (5.8)	3 (10.3)	0.81
Previous late miscarriage, n (%)	6 (0.6)	1 (3.4)	0.19
Previous curetage, n (%)	182 (20.9)	9 (34.6)	0.14
**Maternal outcomes**			
Gestational age at delivery, mean (SD)	39.4 (1.9)	39.8 (1.3)	0.22
Preterm delivery < 37 weeks	54 (5.7)	0	0.40
Spontaneous preterm delivery			
< 37 weeks	37 (3.9)	0	0.62
< 32 weeks	8 (0.8)	0	1.0
Late miscarriage from 16 to 21 weeks	3 (0.3)	0	1.0
Hospitalization for preterm labor	51 (5.5)	1 (3.6)	1.0
Induced preterm delivery < 37 weeks	17 (1.8)	0	1.0
Gestational hypertension	48 (5.0)	0	0.39
Preeclampsia	34 (3.6)	0	0.62
Intra-utero fetal death	5 (0.5)	0	1.0
Mode of delivery^a^			
Vaginal	705 (74.8)	18 (62.1)	0.24
Cesarean during labor	132 (14.0)	7 (24.1)	
Planned cesarean	103 (10.9)	4 (13.8)	
**Neonatal outcomes**			
Birth weight, median (IQR)	3350 (3030–3670)	3590 (3220–3795)	0.06
Small for gestational age (<10th percentile), n (%)	70 (7.4)	2 (6.9)	1.0
Birthweight <1500 g, n (%)	9 (1.0)	0	1.0
Neonatal infection, n (%)	12 (1.3)	0	1.0
Transfer to neonatal intensive care unit, n (%)	75 (8.0)	1 (3.5)	0.72
Respiratory distress syndrome, n (%)	34 (3.6)	1 (3.5)	1.0
Bronchopulmonary dysplasia, n (%)	1 (0.1)	0	1.0
Intraventricular hemorrhage, n (%)	1 (0.1)	0	1.0
Perinatal death, n (%)	1 (0.1)	0	1.0

Percentages are for total available data (missing data < 10% only for sociodemographic and previous medical history data);

^a^For this line and above, n = 940 (exclusion of cases of intra-utero fetal death and delivery from 16 to 21 weeks.);

BMI, body mass index; IQR, interquartile range; Comparisons between quantitative variables involved Kruskall Wallis test. For categorical variables, chi-square or Fisher exact test was used as appropriate.

Finally, all amniotic-fluid samples were screened again by using broad-spectrum PCR and sequencing targeting 16S ribosomal RNA to detect other bacteria. Five women were positive for *Sphingomonas sanxanigenes*, *Acinetobacter baumannii*, *Bacillus niabensis*, *Acinetobacter johnsonii* and *Streptococcus oralis*, all considered probable contaminants. None of these women delivered before 37 weeks.

## Discussion

Although we were unable to complete our trial because of a low recruitment, our main findings are that bacterial colonization in second-trimester amniotic fluid is rare. Indeed, our use of highly sensitive and specific techniques revealed a rate of 3% colonization of amniotic fluid by *Mycoplasma* and/or *Ureaplasma* spp. in second-trimester asymptomatic women. However, this amniotic fluid colonization was not associated with preterm birth or other adverse maternal or neonatal outcomes.

### Strengths and weaknesses

Our study’s main weakness is that we failed to recruit enough cases to test our primary hypothesis—that the use of antibiotics to reduce PCR-positive *Ureaplasma* or *Mycoplasma* spp. infection in amniotic fluid would reduce the incidence of preterm birth. Indeed, we found a marked decrease in number of inclusions because of generalized first-trimester screening for Down syndrome that led to a strong reduction in rate of second-trimester amniocentesis [20]. Moreover, the number of PCR-positive women was much lower than the 17% expected—3% [5, 6]. Therefore, we stopped the study prematurely. As well, we included only women screened for Down syndrome who were therefore not representative of the overall population of women giving birth. However, the rate of preterm delivery (spontaneous or induced) agreed with French national rates, and we do not think that this study feature limits the scope of our results.

Our study has many strengths. This is the largest study to assess the association of amniotic-fluid colonization by *Mycoplasma* and/or *Ureaplasma* spp. and preterm delivery in second-trimester asymptomatic women and to explore the bacterial colonization of asymptomatic second-trimester women. Moreover, its multicentric and prospective design reinforces the strengths of our results. However, DNA but not live cells of mycoplasmas and ureaplasmas was detected in our study, and we cannot exclude that the detected mycoplasmal DNA in the amniotic fluid could come from maternal blood across the placenta.

The real-time PCR used to detect *Ureaplasma* spp., *M*. *hominis* and *M*. *genitalium* is highly sensitive and specific. Indeed, real-time PCR is much more sensitive than culture for detecting these microorganisms and the 3 PCR assays were published and showed very good sensitivity and specificity; the limit of detection was < 5 to 7 genome copies depending on the assay [12–14]. Furthermore, an extraction and amplification control was used for the 3 assays to confirm the quality of DNA extracts. These 3 PCR assays are routinely used in the French expert center for detecting human mycoplasmas. Thus, the low rate of amniotic-fluid positivity on PCR should not be related to the sensitivity of the detection methods used.

Finally, and to investigate whether women who delivered prematurely were positive for other bacteria during the second trimester of pregnancy, we also tested all amniotic-fluid samples with a 16S rDNA PCR, and none of the women who delivered prematurely was positive on 16S rDNA PCR.

### Interpretation

During 2003 to 2006, 3 studies reported that amniotic fluid in second-trimester asymptomatic women may be colonized by *M*. *hominis* and *Ureaplasma* spp. and that colonized women had an increased rate of preterm delivery [5, 6, 7]. The prevalence of these bacteria, detected by a specific end-point PCR or by 16S rDNA PCR [6, 21] in amniotic fluid, was 11% for *Ureaplasma* spp. and 6% for *M*. *hominis* between 15 and 20 weeks. One of those studies focused on *Ureaplasma* spp. in 254 asymptomatic women with amniotic fluid collected by amniocentesis performed for chromosomal analysis [5]. Overall, 24% of PCR-positive women gave birth before 37 weeks versus 0.4% of PCR-negative women (p <0.001). The same analysis was carried out for *M*. *hominis* [6]. The prevalence of *M*. *hominis* in the latter study was 6.4% among 456 patients. In total, 10.4% of the PCR-positive women gave birth prematurely versus 1.9% for PCR-negative women (p = 0.02). The main flaws of these studies were that they were retrospective and conducted by the same team, therefore needing external confirmation by others.

Recently, new data contradicted these findings [22]. Payne et al. tested second-trimester amniotic-fluid cytokine levels and *Ureaplasma* spp. colonisation in 480 Chinese and 492 Australian women. *Ureaplasma* spp. was detected in only 2 Chinese women who delivered preterm [23]. Rowland et al. prospectively investigated amniotic fluid from 344 asymptomatic women recruited in mid-pregnancy for detecting microbial DNA at the time of amniocentesis. *U*. *urealyticum*, *U*. *parvum*, *M*. *hominis* and *M*. *genitalium* were not detected in amniotic fluid. Three women delivered at 30, 31 and 33 weeks in this population. Our study agrees with these results and shows that amniotic-fluid bacterial colonization by *Mycoplasma* or *Ureaplasma* spp. or other bacteria is rare in asymptomatic women.

Several hypotheses can explain these differences with previous studies reporting high rates of *Mycoplasma* or *Ureaplasma* spp. colonization in amniotic fluid of second-trimester women. Indeed, the design of the previous studies was retrospective, they were based on a much smaller number of women, and amniotic-fluid samples were stored after amniocentesis and were not from a prospective study with specific care (especially to avoid any bacterial contamination) [5, 6, 7]. The PCR results from previous studies may have included many false-positive results, specifically from contamination after amniocentesis.

One surprising finding is that *Mycoplasma* or *Ureaplasma* spp. colonization was not associated with preterm birth. This finding is not explained by the fact that 11 women received josamycin. Indeed, 18 women did not receive treatment, and none of the 29 women who were PCR positive had a preterm delivery. The other hypothesis might be that the techniques used are very sensitive and that the very low concentration detected is not able to induce local inflammation and a subsequent preterm delivery. Higher concentrations of bacteria occurring later in the pregnancy or in cases of preterm labor or preterm rupture of membranes may be a cofactor, increasing local inflammation and therefore the risk of preterm birth.

However, intra-amniotic infection by mycoplasmas and ureaplasmas may not be the main factor leading to preterm birth. Subclinical infection has been widely considered a significant etiological factor in the pathogenesis of inflammatory preterm birth, but the source of infection and the gestational window when it is acquired remain unclear. Colonization with low-virulence microorganisms, such as *Ureaplasma* spp. (*U*. *parvum* and *U*. *urealyticum*) and *M*. *hominis*, ascending from the lower genital tract may be the precursor to intra-amniotic subclinical infection, provoking, via metalloprotease and cytokine production, cervical changes in uterine contraction and rupture of membranes leading to preterm birth [3, 24–27]. Genital mycoplasmas commonly isolated in amniotic fluid in cases of preterm labor or PPROM have been implicated in this process. However, whether the microorganisms present are themselves pathogenic is unclear: they may be susceptibility markers or have an adjunct role in the inflammatory preterm birth cascade. Indeed, *Mycoplasma* or *Ureaplasma* spp. are part of the normal vaginal flora in pregnant women [28, 29]. The amniotic-fluid colonization in cases of preterm labor or PPROM may be attributed to cervical dilation or membrane rupture rather than cause preterm labor or PPROM. Although the presence of inflammation has been considered a consequence of microbial presence, the host inflammatory response may, by its effect on cervical dilation or rupture of membranes, favor microbial colonization and invasion. Some recent data favor this hypothesis. Indeed, recent studies using mass spectrometry of both amniotic-fluid culture and broad-range PCR have found that sterile intra-amniotic inflammation is as common as microbial-associated intra-amniotic inflammation in women with preterm labor or PPROM, which suggests that sterile inflammation is more common and more closely associated with preterm birth than microbial-associated inflammation [30, 31]. Moreover, Gervasi et al. reported an increase in markers of inflammation in mid-trimester samples that in some cases was associated with spontaneous preterm birth, although samples did not contain microorganisms detectable with culture techniques [32]. Of note, sterile inflammation ignores any inflammation that may be present due to a viral agonist. Indeed, viruses generally go unnoticed in a diagnosis of infection because they are usually not screened. In addition, the presence of microbial DNA (without live organisms) may invoke an inflammatory response.

### Conclusion

Although low amniotic-fluid colonization rate caused a premature interruption of the trial, our study shows that bacterial colonization of second-trimester amniotic fluid is rare. Colonization by *Mycoplasma* or *Ureaplasma* spp. occurred in 3% of cases and was not associated with risk of preterm birth in this population.

## Supporting information

S1 Table Broad-spectrum PCR and sequencing. (DOCX)

S1 Checklist Consort check list. (DOC)

S1 File English translation of the study protocol. (DOCX)

S2 File Study protocol. (PDF)

S3 File Instruction for use of the Kit Ref. No.: DICD-XX-L100. (PDF)
