# Supplementary material for: Antibiotics for amniotic-fluid colonization by Ureaplasma and/or Mycoplasma spp. to prevent preterm birth: A randomized trial
Source: PLoS One. 2018 Nov 7;13(11):e0206290. doi: 10.1371/journal.pone.0206290 (PMC6221323; doi:10.1371/journal.pone.0206290)
Supplement: S1 Table — (DOCX) [file pone.0206290.s001.docx]

**Table 1S.** Broad-spectrum PCR and sequencing.

| **Targeted sequences** | **Primers** | **Sequences (5’ to 3’)** | **Method** |
| --- | --- | --- | --- |
| 16S rRNA | Fd1 | AGA GTT TGA TCC TGG CTC AG | PCR |
|  | Rp2 | ACG GCT ACC TTG TTA CGA CTT | PCR |
|  | 536F | CAG CAG CCG CGG TAA TAC | Sequencing |
|  | 536R | GTA TTA CCG CGG CTG CTG | Sequencing |
|  | 800F | ATT AGA TAC CCT GGT AG | Sequencing |
|  | 800R | CTA CCA GGG TAT CTA AT | Sequencing |
|  | 1050F | TGT CGT CAG CTC GTG | Sequencing |
|  | 1050R | CAC GAG CTG ACG ACA | Sequencing |
