# Supplementary material for: Antibiotics for amniotic-fluid colonization by Ureaplasma and/or Mycoplasma spp. to prevent preterm birth: A randomized trial
Source: PLoS One. 2018 Nov 7;13(11):e0206290. doi: 10.1371/journal.pone.0206290 (PMC6221323; doi:10.1371/journal.pone.0206290)
Supplement: S1 File — (DOCX) [file pone.0206290.s003.docx]

**English translation of the study protocol**

2 Objective of the study

  To test the effectiveness of antibiotic therapy with Josamycin in case of positive PCR for Ureaplasma spp. and / or Mycoplasma hominis to reduce spontaneous prematurity in asymptomatic patients who perform amniocentesis in the second trimester.

3 Type of study

This is a therapeutic trial in a multicenter randomized placebo double blind study.

Seven maternities with a prenatal diagnosis center and a bacteriology laboratory will participate in the study.

Monitoring and randomization will be provided by the URC Henri Mondor University Hospital.

Packaging of the drug and placebo will be provided by a Bayer laboratory. Lot numbering, dispatch to the central pharmacies of the investigating centers, returns, destruction and stability studies of the active ingredient will be carried out by the AGEPS clinical trial unit.

The methodological and statistical aspect is provided by the Epidemiological Research Unit on the Health of Women and Children (INSERM U 149).

4 Population studied

The study population will be composed solely of patients who have amniocentesis during pregnancy in order to perform a prenatal diagnosis. Indeed, there is no invasive procedure performed for the study and no additional risk of bleeding for the patient. Only 3 ml of amniotic fluid will be reserved for the study on the total quantity taken normally in the patient who is variable (between 15 and 40 ml) according to the objectives of the prenatal diagnosis. This additional 3 ml has no impact on the risk of fetal loss secondary to amniocentesis that is attributable to the puncture itself and not to the amount removed.

It is possible that the study population is different from the general population. In fact, patients with amniocentesis are older and present a risk of obstetric complications during pregnancy, which may be different from younger women, particularly with regard to hypertension and preeclampsia. Nevertheless, there is no evidence of an increased risk of premature birth due to age.

Finally, amniocentesis leads to an increased risk of fetal loss estimated at 0.6 to 1% during pregnancy, 32 but this does not distort comparability between groups since all patients have amniocentesis.

5 Inclusion criteria

 Major patient (≥ 18 years old)

 Speaking and understanding French

 Patient affiliated with social security or a similar scheme

 Having an indication of amniocentesis for karyotype analysis and normal morphological ultrasound (apart from minor signs of trisomy 21)

 With amniocentesis a clear amniotic fluid (uncontaminated by maternal blood)

 whose gestational age is between 15 and 20 weeks

 Not having known allergy to macrolides

 Patient followed during her pregnancy in an investigative center

 giving informed and signed consent

6 Exclusion criteria

 Minor patient

 Not understanding French

 Having an allergy to macrolides

 Having a multiple pregnancy

 Morphological abnormality on ultrasound (apart from minor signs of trisomy 21)

 Patient refusing to participate in the trial for any reason

 Known intolerance to Lactose

7 Factor studied: PCR for Mycoplasma hominis and Ureaplasma spp.

In order for the PCR procedure to be homogeneous, it will be completely performed in the bacteriology laboratory of Cochin. This has a direct implication on the centers that can be included in the study. The distance plays indeed a major role, an important distance of the centers would prevent a fast routing essential for the study. On the other hand this would require special transport conditions (freezing at -80 ° C) that would make the costs very high.

The search for Mycoplasma hominis and Ureaplasma spp. (U. parvum and U. urealyticum) will be performed by PCR on the amniotic fluid after extraction of the DNA. Briefly, the DNA will be extracted from 200 μl of each sample using the Qiagen extraction kit. The PCRs will be performed using primers specific for highly conserved areas found in the urease gene for Ureaplasma spp. and in the 16S RNA gene for M. hominis (Table VII). The sequences of these primers are described, evaluated and published in several studies 24, 26-28.

The specificity of the amplification product will be evaluated by an ELISA technique during which the amplicons will be denatured, biotinylated and evaluated by measuring the optical density of a colorimetric reaction. The amplified products will also be characterized by their size, after migration on agarose gel containing BET. For Mycoplasma hominis the fragment obtained will also be subjected to a digestion in enzymatic allowing a more specific identification. The PCR technique may also allow the distinction between the two recently identified Ureaplasma species (U. parvum and U. urealyticum).

8 Antibiotics used

The desired site of action of antibiotics is the deciduous, the chorion and the amnion. Indeed, a positive PCR in the amniotic fluid essentially testifies to an attack of these choriodecidural tissues and those should be preferentially concerned by the antibiotherapy.

The target bacteria for antibiotic therapy are Mycoplasma hominis and Ureaplasma spp.

Tetracyclines, macrolides and related drugs (lincosamides including clindamycin) and fluoroquinolones are the main families of antibiotics active against these bacteria.33 The antibiotics that can be used are macrolides and lincosamides because tetracyclines and fluoroquinolones are contraindicated during pregnancy. pregnancy and in children.

Ureaplasma spp. is sensitive to macrolides but resistant to lincosamides. In contrast, Mycoplasma hominis is resistant to 14- and 15-carbon macrolides such as erythromycin but is sensitive to certain 16-carbon macrolides (josamycin and midecamycin) and lincosamides. Among the different macrolides, josamycin represents the best compromise for its activity on the two bacterial species with minimum inhibitory concentrations for Mycoplasma hominis <1mg / L and for Ureaplasma spp. <2mg / L (Table VIII) 34.

A dosage of two grams per day of josamycin for 10 days would allow sufficient intratissue concentrations to be active on mycoplasmas for a sufficiently long time to reduce the risk of recurrence.

12 Criteria for judgment

12.1 Primary judgment criterion

Occurrence of premature delivery between 22 and 37 weeks

12.2 Secondary judgment criteria.

12.2.1 Obstetrical

Prenatal:

• Late miscarriage occurred between 16 and 22 weeks

• Premature delivery ≤ 34, 32, 28 SA.

• Hospitalization for the threat of premature labor

• Number of days of hospitalization for threat of preterm birth

• Premature rupture of the membranes before 37 SA

• Occurrence of chorioamnionitis defined by two of the following criteria: maternal temperature> 38 ° C, uterine contractions, foul leucorrhea, fetal tachycardia> 160 bpm, CRP> 10 mg / L.

During work :

• Hyperthermia> 38 ° C

• Fetal tachycardia> 160 bpm

In the postpartum:

• Hyperthermia> 38 ° C for more than 24 hours

• Need for antibiotic treatment for more than 48 hours

Neonatal 12.2.2

12.2.2.1 Neonatal mortality

• Early neonatal mortality from D0 to D6

• Late neonatal mortality from D7 to D28.

12.2.2.2 Neonatal morbidity

Immediate neonatal condition

Immediate neonatal status will be judged on the Apgar score, cord pH and transfer to neonatal resuscitation or ICU.

Neonatal infection:

It is most often caused by group B streptococci or Escherichia coli and may increase the risk of ventricular leukomalacia and bronchopulmonary dysplasia when it follows inflammation in utero 35. Clinical expression of infection in the new is not polymorphic. Any anomaly of the clinical examination that does not fit into evidence in another pathology will be considered as a sign of infection: respiratory distress not related to a resorption disorder or hyaline membrane disease, haemodynamic disorders, abnormality neurological examination.

The biological parameters studied will be, peripheral cultures, central crops, CRP (positive if greater than or equal to 10 mg / l), leukocytosis (positive if greater than 25000 or less than 5000) .These results are based on examinations systematically performed in the newborn. No further review will be conducted for this research on newborns.

We will consider as:

• Certain infection: Peripheral or central positive specimens and elevated CRP greater than 10 mg / l

• A probable infection: An abnormal clinical examination attributable to an infection or context of maternal chorioamnionitis and a CRP elevation greater than 10 mg / l.

Failure to meet the criteria listed above will cause the newborn to be uninfected.

Respiratory morbidity:

• Immediate:

• Transient respiratory distress

• Hyaline membrane disease

• Late :

• Oxygen dependence at 28 days

• Oxygen dependence at 36 SA

Cerebral morbidity

• Intra-ventricular hemorrhage grade 1-2-3-4 according to the criteria of Papile 36

• Periventricular leukomalacia

Digestive morbidity

• Occurrence of ulcerative necrotizing enterocolitis according to Bell's classification 37.

3 Practical realization of the study (Appendix I)

13.1 Inclusion of Patients and Inclusion File Filling: Role of Investigator and Clinical Research Technician

Participation in the research will be proposed to patients who will present themselves in the investigative departments for the realization of an amniocentesis between 15 and 20 AS for a prenatal diagnosis. All criteria will be verified by the investigating physician of the center.

Patients who meet all the inclusion criteria will be offered the opportunity to participate in the PREMYC trial.

The course of the study will be explained to the patient (information leaflet). The physician in charge of amniocentesis will collect a part of the informed consent signed by himself and by the patient. It will be made clear to the patient that she may refuse to participate in the trial, or withdraw her consent during the course of the study, without her care and relations with her doctor being affected.

At inclusion, the physician will complete the patient's inclusion sheet on a secure electronic CRF ("cleanweb" software).

This initial seizure will signal the inclusion of the patient in the clinical research technician who will then be responsible for entering patient data during the study.

The use of this computerized electronic FIU directly accessible via the Internet will facilitate interaction between the clinical centers, the bacteriology laboratory, the Henri Mondor CRU and the DRCD. In addition, a large part of the seizure can be done centrally by the clinical research technician. Finally, much of the monitoring by the Clinical Research Associate can be done centrally.

13.2 Anonymization

 The study data will be anonymized, with each patient being identified by:

- the first letter of her maiden name

- the first letter of his first name

- the number of the center

- the inclusion number

These identification parameters will appear on the samples and the eCRF.

13.3 Sending the sample to the bacteriology laboratory

The amniotic fluid samples for research (PCR for Mycoplasma hominis and Ureaplasma spp) will be stored at 4 ° C in a specific medium for a maximum of 3 days and delivered by the APHP coureur to the bacteriology laboratory. Cochin hospital where they will be received by the laboratory technician.

13.4 Selection of patients for the therapeutic trial

The result of the PCR will be transmitted the same day by fax to the investigating doctor (and the URC Henri Mondor).

If the PCR is negative, the patient will be informed of the result by telephone. The doctor will inform her that she will not participate in the therapeutic trial but that the follow-up of her pregnancy will be carried out in a traditional way and that the data collected during this follow-up will be used for research purposes (until delivery and her maternity leave). The patient will be reviewed by the investigating physician during scheduled visits as part of the normal follow-up of her pregnancy. Since no visit is done specifically for the research, it does not entail any additional cost for the patient.

If the PCR is positive for Ureaplasma spp. and / or Mycoplasma hominis, the patient will be contacted by the clinical research technician who will propose an appointment with the doctor to announce this result to him and propose him the batch of treatment.

The doctor will make sure that the patient is willing to continue the research and participate in the therapeutic trial.

If the patient does not wish to participate in the therapeutic trial, the patient will be out of the study. The monitoring of her pregnancy will be ensured according to the current practices of the service, and in the best interest for the patient and her future child.

If the patient wishes to continue her participation, an appointment will be offered for a treatment dispensation visit (if telephone contact). During this visit, the patient will be randomized in the group (Josamycin) or in the group (Placebo). Neither the doctors nor the patients will know in which group they belong. Patient travel expenses will be borne by the research sponsor.

3.5 Randomization and Treatment Administration

The randomization will be done according to a stratified randomisation list on the centers, prepared by the URC Henri Mondor.

The randomization will be done directly online from the cleanweb software.

The batch of treatment will then be given to the patient by the pharmacy, on a specific order of the investigator (Josamycin or placebo, at a dosage of 1 gram morning and evening orally for 10 days).

A blind survey card will be given to patients. (See chapter on the lifting of blindness)

The treatment batches will be distributed in the pharmacies of the investigative centers by the AGEPS. They will be labeled in accordance with the legislation in force for drugs intended for therapeutic trials. In order to guarantee blindness, josamycin or placebo treatment batches will look identical

13.6 Monitoring treatment adherence

A follow-up and patient recall procedure will be performed to monitor adherence to treatment. A telephone call-back will take place 15 days after the beginning of the treatment and will check with a short questionnaire the compliance and the absence of side effects related to the treatment (Appendix II)

The patient will also be asked to return the blank, opened or empty treatment pack at her next consultation. Consultation visits will be monthly until delivery. These boxes will be kept in the pharmacies of the centers until the end of the research.

13.7 Reporting of patients included in the medical file

The inclusion of the patient in the study will be marked by a pastille pasted on the cover page of her clinical file. . If the patient is included and randomized a second label will be placed on the medical record to indicate that she is taking either an antibiotic or a placebo.

13.8 Patient follow-up

The follow-up of all PCR + / PCR- patients will be recorded under the responsibility of the investigator by the clinical research technician.

In order to check for possible confounding factors, data concerning the management, the evolution of pregnancy, delivery and the characteristics at birth of the newborn will be collected.

Maternal follow-up will be carried out until the maternity leave.

The follow-up of premature newborns will be carried out until a corrected age of 36 weeks.

The follow-up of the newborns will be realized until the exit of the maternity.

Data on pregnancy outcome after inclusion in the study will be collected.

Data regarding vaginal sampling between 35 and 37 weeks will be collected.

Data concerning the prescription of antibiotics during pregnancy will be collected.

Data on delivery and neonatal follow-up before leaving the maternity ward will be collected.

13.9 Data collection

The data will be collected as the study progresses directly on the computer in the electronic CRF using the "cleanweb" software.

The data will be collected directly by the clinical research technician as the patient is delivered.

16.1 Number of subjects needed

We estimated the risk of preterm delivery to be 17% in the case of positive amniotic fluid PCR for Ureaplasma spp. or Mycoplasma hominis, (average assumption between 10 to 24% corresponding to published figures). In order to be able to show a reduction in the risk of preterm delivery by 50%, it is necessary to include 238 women in each group (α = 0.05, 1-ß = 0.80).

The prevalences published in the second trimester of positive PCR for Ureaplasma spp. and Mycoplasma hominis vary little according to studies and countries (USA or Switzerland) and are at least 15% by combining the two bacteria 26. The inclusion of 3200 patients who benefit from amniocentesis will achieve the desired number of patients.

16.2 Duration of the study and feasibility

The number of amniocentesis performed over one year in all centers participating in the study is approximately 3750 amniocentesis per year, distributed as follows:

- CHI of Créteil: 400

- Port Royal: 500

- Saint Vincent de Paul: 550

- CHI of Poissy 650

- Rothschild Keychain: 200

- Saint Anthony: 650

- Necker: 200

- Robert Debré: 600

Assuming an inclusion rate in the study of 40% (refusal of patients, twin pregnancies, malformations ...), the number of 3200 patients could be reached in 30 months on all centers that have agreed to participate in the study. study. The follow-up of patients and newborns is 6 months after the date of inclusion. The total duration of the study would be 36 months. The number of patients lost to follow-up should in principle be very low because they are pregnant patients who receive very regular follow-up and for whom the delivery will necessarily take place in a maternity ward.

16.3 Data Analysis

The data capture will be carried out as and when under the responsibility of the investigator through the software "cleanweb". The computer file will be processed under STATA (College Station, TX, USA)

Data analysis will be done in INSERM unit U149 by F Maillard and G Kayem.

The study will distinguish 3 groups of patients according to the studied factor and the prescription of an antibiotic treatment: with a negative PCR, with a positive PCR without antibiotic treatment, with a positive PCR with antibiotic treatment.

These groups will be compared to each other for

• Clinical criteria collected at baseline: maternal age and level of education, obstetrical history, gestational age at baseline.

• Criteria collected during pregnancy: number of consultations, treatment, concomitant treatments, protocol outputs, adverse events and unblinding.

Then, an intention to treat the existence of an "antibiotic" effect in the form of a difference, in the case of a positive PCR, between the "placebo" group and the "josamycin" group for the main endpoint and the criteria of secondary judgments.

Comparisons will use non-parametric statistical tests whenever staffing or distribution constraints require it. For categorical variables, the chi2 test and the Fisher test will be used. Variance analysis and Kruskal and Wallis nonparametric testing will be used for continuous variables.

The effects will be measured as relative risk, with their 95% confidence interval.
