# Supplementary material for: Antibiotics for amniotic-fluid colonization by Ureaplasma and/or Mycoplasma spp. to prevent preterm birth: A randomized trial
Source: PLoS One. 2018 Nov 7;13(11):e0206290. doi: 10.1371/journal.pone.0206290 (PMC6221323; doi:10.1371/journal.pone.0206290)
Supplement: S2 File — (PDF) [file pone.0206290.s004.pdf]

REDUCTION DE LA PREMATURITE SPONTANEE : IMPACT D'UN TRAITEMENT ANTIBIOTIQUE EN CAS DE PCR POSITIVE POUR *UREAPLASMA* SPP. ET/OU *MYCOPLASMA HOMINIS* DANS LE LIQUIDE AMNIOTIQUE

Projet PREMYC

Investigateur Coordonnateur:
Docteur Gilles Kayem
Service de Gynécologie Obstétrique
CHI de Créteil
40 avenue de Verdun
94000 Créteil

Promoteur :
Assistance Publique-Hôpitaux de Paris
Département de la Recherche Clinique et du Développement
Carré historique de l'Hôpital saint-Louis
1 avenue Claude Vellefaux
75010 Paris

Page de SIGNATURE D'UN PROTOCOLE de recherche biomédicale
par l'investigateur COORDONNATEUR et le représentant du PROMOTEUR

Recherche biomédicale N°P060216 code : PREMYC

Titre : « Réduction de la prématurité spontanée : impact d'un traitement antibiotique en cas de pcr positive pour *ureaplasma* spp. et/ou *mycoplasma hominis* dans le liquide amniotique »

Version N° 2 du : 15/06/07

L'investigateur coordonnateur :
Nom et prénom, titre Dr Kayem Gilles
adresse

L'investigateur coordonnateur autorise également la diffusion de son identité et de ses coordonnées dans le répertoire des recherches biomédicales

Date : ……………/………/………..

Signature :

Le promoteur :
Dr Olivier CHASSANY
Assistance publique – hôpitaux de Paris
Délégation Interégionale à la recherche clinique
Hôpital Saint Louis
75010 PARIS

Date : ……………/………/………..

Signature :

NB : cette version correspond au texte du protocole et annexes adressés au CPP et à l'autorité compétente respectivement pour avis et demande d'autorisation.
Si ensuite une autre version est rédigée suite à des modifications, il faut refaire le circuit des signatures afin d'être toujours à jour des versions du protocole actif.

<u>Comité de pilotage de l'étude</u> :
Pr Gérard Bréart, Pr Dominique Cabrol, Dr Robert Cohen, Dr Claude Danan, Pr François Goffinet, Pr Bassam Haddad, Pr Claire Poyart, Shohreh Azimi ,Noël Boudjema, Christophe Aucan

<u>Investigateur coordonnateur</u> : Dr Gilles Kayem
Service de Gynécologie-Obstétrique CHI de créteil
40, avenue de Verdun 94000 créteil
Tél : 01 45 17 55 74
INSERM Unité 149
Hôpital Saint Vincent de Paul
82, avenue Denfert Rochereau 75014 Paris

<u>Investigateurs associés</u> :

Service de gynécologie obstétrique du CHI Créteil (chef de service : Pr Paniel):
Pr B Haddad
Service de Gynécologie-Obstétrique
40, avenue de Verdun 94000 créteil
Tél : 01 45 17 55 43
Fax : 01 45 17 55 42

Maternité Port Royal, groupe Hospitalier Cochin - Saint Vincent de Paul - La Roche Guyon, APHP, Paris (chef de service : Pr Cabrol):
Dr T Schmitz
123, bd de Port Royal, 75679 Paris cedex 14
Tél : 01 58 41 21 42
Fax : 01 58 41 29 82

Service de gynécologie obstétrique de Saint Vincent de Paul, groupe Hospitalier Cochin-Saint Vincent de Paul-La Roche Guyon, APHP, Paris (chef de service : Pr Tournaire) :
Dr F Lewin
82, avenue Denfert Rochereau 75014 Paris
Tél : 01 40 48 81 42
Fax : 01 40 48 81 41

Service de gynécologie obstétrique de St Antoine, APHP, Paris
(chef de service : Pr Milliez) : Pr B Carbonne
SAINT-ANTOINE
184, rue du Faubourg Saint-Antoine 75012 Paris
Tél : 01 49 28 28 76
Fax : 01 49 28 29 74

Service de gynécologie obstétrique du CHI de Poissy (chef de service : Pr Ville) : Dr P Rozenberg
10, rue Champ Gaillard 78300 Poissy
Tél : 01 39 27 52 57
Fax : 01 39 27 44 79

Service de gynécologie obstétrique de l'hôpital Necker, APHP, Paris (chef de service : Pr Dumez) : Dr A Benachi
149, rue de Sèvres 75015 PARIS
Tél : 01 44 49 40 30
Fax : 01 44 49 40 31

Service de gynécologie obstétrique de l'hôpital Robert Debré
Pr Jean François OURY
48, Bd Serrurier 75019 Paris
Tél : 01 40 03 21 70
Fax : 01 40 03 24 80

Service de gynécologie obstétrique de l'hôpital Trousseau, APHP, Paris (chef de service : Pr Benifla) : Dr JM Jouannic
26, avenue du Docteur Arnold-Netter**75012 Paris**
Tél : 01 40 19 37 25
Fax : 01 40 19 37 19

Laboratoire de bactériologie, Cochin, groupe Hospitalier Cochin - Saint Vincent de Paul - La Roche Guyon, APHP, Paris (chef de service : Pr C Poyart) : Pr C Poyart
82, avenue Denfert Rochereau 75014 ParisTél : 01 58 41 15 61
Fax :01 58 41 15 48

INSERM U 149 (Pr G Bréart) : **Pr F Goffinet**
Hôpital Saint Vincent de Paul
82, avenue Denfert Rochereau 75014 ParisTél : 01 58 41 20 69
Fax : 01 58 41 21 05

Biostatisticien : Unité de Recherche épidémiologique sur la santé des femmes et des enfants (INSERM U 149) : Françoise Maillard
Hôpital Saint Vincent de Paul
82, avenue Denfert Rochereau 75014 ParisTél : 01 58 41 20 69
Fax : 01 58 41 21 05

Unité de Recherche Clinique : **Hôpital Henri Mondor**
51, avenue du Mal de lattre de Tassigny
94000 Créteil
Tél : 01 49 81 37 52/37 98
Fax : 014981 37 99

Unité Essais Clinique de l'AGEPS :
7, rue du fer à moulin 75006 Paris
Tél : 01 46 69 13 13

Promoteur :
Assistance Publique-Hôpitaux de Paris
Département de la Recherche Clinique et du Développement
Carré historique de l'Hôpital saint-Louis, 1 avenue Claude Vellefaux
75010 Paris
Tél : 01 44 84 17 23
Fax : 01 44 84 17 99

Résumé du projet

Position du problème : L'infection serait la cause de 40 % des accouchements prématurés spontanés. L'hypothèse physiopathologique admise est une ascension précoce de bactéries présentes dans les voies génitales basses vers la décidue, les membranes fœtales (chorion et amnios) puis le liquide amniotique. Ces bactéries sont responsables d'une réaction inflammatoire à l'interface foetomaternelle caractérisée par la production de cytokines pro-inflammatoires et d'agents procontracturants (prostaglandines, ocytocine) par la décidue et les membranes. Ces médiateurs provoquent des contractions utérines, une maturation du col utérin, une rupture des membranes puis un accouchement prématuré. Plusieurs publications récentes montrent d'une part que *Mycoplasma hominis* et *Ureaplasma* spp. sont les bactéries les plus fréquemment retrouvées dans le liquide amniotique au second trimestre de la grossesse et d'autre part qu'une PCR positive pour ces bactéries est associée à un accouchement prématuré. Une hypothèse vraisemblable serait que *Mycoplasma hominis* ou *Ureaplasma* spp. provoquent un accouchement prématuré en infectant les membranes fœtales et la décidue puis en activant le système immunitaire et la production de cytokines pro-inflammatoires. Ces bactéries sont accessibles à un traitement antibiotique. Néanmoins, aucun essai randomisé n'a été effectué pour déterminer si un traitement antibiotique pouvait diminuer la prématurité spontanée en cas de PCR positive dans le liquide amniotique.

Objectif de l'étude : Tester l'efficacité d'un traitement antibiotique (Josamycine) en cas de PCR positive pour *Ureaplasma* spp. et/ou *Mycoplasma hominis* au second trimestre sur le risque d'accouchement prématuré.

Population et méthode : Etude multicentrique prospective randomisée contre placebo en double insu. Les patientes qui bénéficieront d'une amniocentèse entre 15 et 20 semaines d'aménorrhées pour un diagnostic prénatal se verront proposer l'inclusion dans l'étude. Une détection de *Mycoplasma hominis* et *Ureaplasma* spp. par PCR sera réalisée dans le liquide amniotique.

En cas de PCR positive, les patientes se verront proposer d'entrer dans un essai destiné à tester l'efficacité d'un traitement antibiotique contre placebo sur la diminution du taux d'accouchement prématuré.

Le traitement antibiotique proposé sera la josamycine à la posologie de 2 grammes par jour par voie orale pendant 10 jours.

Les patientes PCR négatives seront suivies pendant leur grossesse, jusqu'à l'accouchement et la sortie de maternité de la patiente et de son enfant.

Critère de jugement principal : Accouchement prématuré entre 22 et 37 semaines d'aménorrhée.

Analyse : Elle sera effectuée en intention de traiter en analysant l'effet du traitement antibiotique sur le risque d'accouchement prématuré en cas de PCR positive pour *Ureaplasma* spp. et/ou *Mycoplasma hominis*.

Bénéfices attendus de l'étude : Environ 80 000 amniocentèses sont réalisées tous les ans en France. L'efficacité d'une antibiothérapie permettrait de réduire la prématurité spontanée associée à *Mycoplasma hominis* et/ou *Ureaplasma* spp. dans ce groupe de patientes.

Nombre de sujets nécessaires : 238 patientes ayant une PCR positive doivent être incluses dans chaque bras de traitement ($\alpha=0.05$, 1-ß = 0.80). 3200 patientes doivent être dépistées pour inclure 476 patientes avec une PCR positive pour *Ureaplasma* spp. et/ou *Mycoplasma hominis*.

Durée de participation : 7 mois

Durée prévue : Trois ans.

Table des matières

1 JUSTIFICATION DE L'ETUDE 10

1.1 ACCOUCHEMENT PREMATURE ET INFECTION 11
1.2 ASSOCIATION DE *MYCOPLASMA HOMINIS* OU *UREAPLASMA* SPP. AVEC L'ACCOUCHEMENT PREMATURE 13
1.3 ASSOCIATION ENTRE *UREAPLASMA* SPP. ET *MYCOPLASMA HOMINIS* DANS LE LIQUIDE AMNIOTIQUE AU DEUXIEME TRIMESTRE CHEZ DES PATIENTES ASYMPTOMATIQUES ET ACCOUCHEMENT PREMATURE 15
1.4 UTILISATION D'UNE ANTIBIOTHERAPIE PAR LA JOSAMYCINE VERSUS PLACEBO POUR LA PREVENTION DE L'ACCOUCHEMENT PREMATURE. 17
1.5 INTERET D'UN ESSAI THERAPEUTIQUE RANDOMISE CONTRE PLACEBO 18

2 OBJECTIF DE L'ETUDE 19

3 TYPE D'ETUDE 19

4 POPULATION ETUDIEE 20

5 CRITERES D'INCLUSION 21

6 CRITERES D'EXCLUSION 21

7 FACTEUR ETUDIE : PCR POUR *MYCOPLASMA HOMINIS* ET *UREAPLASMA* SPP. 22

8 ANTIBIOTIQUES UTILISES 23

9 EFFETS INDESIRABLES DE LA JOSAMYCINE 24

10 IMPACT DE LA JOSAMYCINE SUR LE RISQUE DE RESISTANCE DE LA FLORE VAGINALE BACTERIENNE 24

11 TRAITEMENTS CONCOMITANTS 25

12 CRITERES DE JUGEMENT 26

12.1 CRITERE DE JUGEMENT PRINCIPAL 26
12.2 CRITERES DE JUGEMENT SECONDAIRES. 26
12.2.1 OBSTETRICAUX 26
12.2.2 NEONATAUX 28

13 REALISATION PRATIQUE DE L'ETUDE (ANNEXE I) 30

13.1 INCLUSION DES PATIENTES ET REMPLISSAGE DE LA FICHE D'INCLUSION : ROLE DE L'INVESTIGATEUR ET DU TECHNICIEN DE RECHERCHE CLINIQUE 30

13.2 ANONYMISATION 31

LES DONNEES DE L'ETUDE SERONT ANONYMISEES, CHAQUE PATIENTE ETANT REPEREE PAR : 31

- LA PREMIERE LETTRE DE SON NOM DE JEUNE FILLE 31

- LA PREMIERE LETTRE DE SON PRENOM 31

- LE NUMERO DU CENTRE 31

- LE NUMERO D'INCLUSION 31

CES PARAMETRES D'IDENTIFICATION FIGURERONT SUR LES PRELEVEMENTS ET LE DOSSIER INFORMATISE. 31

13.3 ENVOI DU PRELEVEMENT AU LABORATOIRE DE BACTERIOLOGIE 31
13.4 SELECTION DES PATIENTES POUR L'ESSAI THERAPEUTIQUE 31
13.5 RANDOMISATION ET ADMINISTRATION DES TRAITEMENTS 32
13.6 CONTROLE DE L'OBSERVANCE DU TRAITEMENT 33
13.7 SIGNALEMENT DES PATIENTES INCLUSES DANS LE DOSSIER MEDICAL 33
13.8 SUIVI DES PATIENTES 34
LE SUIVI DE TOUTES LES PATIENTES PCR+/PCR- SERA CONSIGNE SOUS LA RESPONSABILITE DE L'INVESTIGATEUR PAR LE TECHNICIEN DE RECHERCHE CLINIQUE. 34
13.9 RECUEIL DES DONNEES 34

14 LEVEE DE L'INSU 35

15 CONDITIONNEMENT ET DISTRIBUTION DE L'ANTIBIOTIQUE ET DU PLACEBO 36

16 STATISTIQUES 37

16.1 NOMBRE DE SUJETS NECESSAIRES 37
16.2 DUREE DE L'ETUDE ET FAISABILITE 37
16.3 ANALYSE DES DONNEES 38

17 BENEFICES ATTENDUS DE L'ETUDE 40

18 ASPECTS REGLEMENTAIRES ET LEGISLATIFS 40

18.1 DECLARATION CNIL 40
18.2 DOCUMENTATIONS DE LA RECHERCHE 40
18.3 CONTROLE DE QUALITE ET ASSURANCE QUALITE 41
18.4 GESTION DES EVENEMENTS INDESIRABLES 42
18.4.1 DEFINITIONS : 42
18.4.2 OBLIGATIONS DES INVESTIGATEURS : 42
18.4.3 DECLARATION DES EVENEMENTS INDESIRABLES GRAVES AUX AUTORITES DE SANTE : 43

18.5 TRANSCRIPTION DES DONNEES DANS LE CRF ELECTRONIQUE 44
18.6 AMENDEMENTS AU PROTOCOLE DE LA RECHERCHE 44
18.7 EXTENSION DE LA RECHERCHE 44
18.8 RESPONSABILITE 44
18.9 RAPPORT FINAL DE LA RECHERCHE 45
18.10 PUBLICATIONS ET PROPRIETES DES DONNEES 45
18.11 FORMULAIRE D'INFORMATION AUX SUJETS ET CONSENTEMENT 45
18.12 SOUMISSION AU CPP ET DECLARATION A L'AFSSAPS 46
18.13 AMENDEMENT AU PROTOCOLE DE L'ETUDE ET EXTENSION DE L'ETUDE 46
18.14 DECLARATION CNIL 46
18.15 DOCUMENTATION DE L'ETUDE 46

REFERENCES 52

ANNEXE I : REALISATION PRATIQUE DE L'ETUDE 56

ANNEXE II : CONTROLE TELEPHONIQUE DE L'OBSERVANCE AU TRAITEMENT ET DE LA SURVENUE D'EFFETS SECONDAIRES 57

ANNEXE III : FICHE DE DECLARATION DES EIGS : 58

1 Justification de l'étude

L'accouchement prématuré concernait 7.2 % des naissances en France en 2003 et reste la principale cause de morbidité et de mortalité périnatales [1]. La mortalité néonatale provient dans 60 % des cas d'enfants nés avant 30 SA et la moitié des séquelles neurologiques, cognitives ou respiratoires sont observées en cas de naissance avant 32 semaines d'aménorrhées (SA) [2]. La cause de la prématurité spontanée serait infectieuse dans 40 % des cas [3]. Lorsque l'accouchement survient avant 30 SA, l'association avec l'infection pourrait s'élever à 60 voire 80 %. Cela est d'autant plus préoccupant que l'inflammation associée à l'infection est un facteur de risque de leucomalacie périventriculaire et de dysplasie bronchopulmonaire [4].

L'hypothèse physiopathologique admise est une ascension bactérienne précoce au niveau des tissus choriodéciduaux puis dans le liquide amniotique à partir d'une colonisation vaginale. L'invasion de ces tissus entraînerait la production de cytokines et chémokines pro-inflammatoires, puis la production de prostaglandines. Ces médiateurs provoqueraient une maturation cervicale et des contractions utérines puis une rupture des membranes et un accouchement prématuré. Cette cause d'accouchement prématuré pourrait être prévenue par un traitement antibiotique.

1.1 Accouchement prématuré et infection

L'hypothèse actuelle de la physiologie de l'accouchement prématuré d'origine infectieuse est la suivante [3] : une colonisation bactérienne vaginale contamine par un mécanisme ascendant les membranes fœtales (chorion et décidue) puis le liquide amniotique. Elle peut ensuite se propager au fœtus et provoquer un syndrome inflammatoire fœtal qui est associé à un risque accru de leucomalacies périventriculaires et de dysplasie bronchopulmonaire [5-9].

Le principal support de cette hypothèse est la mise en évidence d'une invasion bactérienne de la cavité amniotique en cas de menace d'accouchement prématuré. La culture du liquide amniotique est positive chez 9 à 20 % des patientes en cas de menace d'accouchement prématuré à membrane intacte et chez 15 à 40 % en cas de rupture des membranes avant terme (tableaux I et II). De plus, une culture positive du liquide amniotique est associée à l'accouchement prématuré, à une chorioamniotite et à l'infection néonatale.

Les bactéries les plus fréquemment retrouvées dans le liquide amniotique sont *Mycoplasma hominis* et *Ureaplasma* spp. et moins souvent *Fusobactérium, Gardenerella vaginalis, Peptostreptococcus* et *Bacteroides* spp. [10]. Ces bactéries sont également retrouvées dans les sécrétions cervicovaginales ce qui permet de supposer que l'invasion bactérienne du liquide amniotique est liée à l'ascension de bactéries d'origine vaginales.

La vaginose bactérienne a été particulièrement étudiée. Il s'agit d'une modification de la flore vaginale normale constituée principalement de lactobacilles et un remplacement de celle-ci par une flore aéroanaérobie polymorphe dont *Gardnerella vaginalis, Mycoplasma hominis* et *Ureaplasma* spp. Plusieurs études récentes montrent une association entre une vaginose bactérienne et l'accouchement prématuré. L'étude la plus récente qui porte sur le plus grand effectif (n = 12997) est celle de Klebanoff et coll [11]. Les auteurs trouvent un odd ratio de 1.4 (1.1-1.6) en cas de vaginose bactérienne, risque similaire à celui retrouvé dans la

preterm prediction study [12]. De plus, ce risque semble augmenté quand le terme du diagnostic de la vaginose bactérienne est précoce au cours de la grossesse [13]. Enfin, le risque d'accouchement prématuré est plus élevé chez les femmes atteintes de vaginose bactérienne et possédant *Mycoplasma hominis* au niveau de leur flore vaginale (OR : 2.1, IC95% : 1.5-3.0) [12].

La physiopathologie de l'accouchement prématuré d'origine infectieuse a été schématisée par Goldenberg (figure 1) [3]. A partir d'une infection des tissus choriodéciduaux se produit une réaction inflammatoire avec une production de cytokines et chémokines pro-inflammatoires associées à un infiltrat de polynucléaires neutrophiles. Ces médiateurs stimulent la production d'agents procontracturants (prostaglandines, ocytocine) et de collagénases (métalloprotéinases) qui sont responsables de contractions utérines, d'une maturation cervicale, d'une rupture des membranes et provoquent finalement un accouchement prématuré [14-18].

L'infection est la cause de 40 % des accouchements prématurés. Elle est liée à une ascension de bactéries provenant des voies génitales qui envahissent la décidue, le chorion et l'amnios puis provoquent une réaction inflammatoire. Celle-ci stimule la production d'agents procontracturants (prostaglandines, ocytocine) et de collagénases (métalloprotéinases) qui provoquent des contractions utérines, une maturation cervicale, une rupture des membranes et finalement un accouchement prématuré.

1.2 Association de *Mycoplasma hominis* ou *Ureaplasma* spp. avec l'accouchement prématuré

Mycoplasma hominis et *Ureaplasma* spp. sont des mycoplasmes qui appartiennent à la classe des Mollicutes. Il en existe plusieurs types qui peuvent coloniser le tractus génital chez la femme (tableau III). Parmi ceux-ci, *Ureaplasma* spp. (*Ureaplasma* biovar 1 et 2 renommés récemment *Ureaplasma urealyticum* et *Parvum*) et *Mycoplasma hominis* peuvent être des pathogènes du tractus urogénital et sont associés à une chorioamniotite et à l'accouchement prématuré (tableau IV) [19]. Ces bactéries à localisation intracellulaire provoquent une infection chronique et une réponse inflammatoire qui peut être infraclinique pendant une longue période.

L'association entre mycoplasmes et chorioamniotite ou accouchement prématuré a été l'objet de nombreux travaux. En cas de menace d'accouchement prématuré avec ou sans rupture des membranes, *Mycoplasma hominis* et *Ureaplasma* spp. sont les bactéries les plus fréquemment retrouvées au niveau du placenta ou du liquide amniotique et sont associées à une chorioamniotite et à l'accouchement prématuré. Kundsin et coll. ont examiné les placentas de 647 femmes qui avaient donné naissance à des enfants prématurés de poids inférieur ou égal à 1500 g [20]. Vingt-huit pour cent des placentas mis en culture hébergeaient *Ureaplasma* spp., ce qui en fait les microorganismes les plus fréquemment isolés à ce niveau. De plus, la détection d'*Ureaplasma* spp. dans le placenta est associée à une chorioamniotite histologique [21].

Plus récemment, l'association entre une PCR positive pour *Ureaplasma* spp. et l'accouchement prématuré ou la morbidité néonatale a été étudiée [22]. Les auteurs ont montré qu'une PCR positive dans le liquide amniotique, en cas de rupture des membranes avant 37 SA, était associée à la production d'IL-6, un accouchement prématuré et un moins bon

pronostic néonatal. De plus, le pronostic des patientes qui avait une PCR positive avec une culture négative du liquide amniotique était similaire de celui des patientes qui avaient une culture positive pour *Ureaplasma* spp.. Ce travail montre que la détection d'un faible inoculat d'*Ureaplasma* spp. par une PCR positive traduit un effet pathogène potentiel équivalent à sa détection par une culture classique.

Plusieurs travaux montrent que *Mycoplasma hominis* et *Ureaplasma* spp. sont associés à l'accouchement prématuré. Une PCR positive du liquide amniotique pour *Ureaplasma* spp. en cas de rupture des membranes avant 37 SA est associée à l'accouchement prématuré et à un moins bon pronostic néonatal.

1.3 Association entre *Ureaplasma* spp. et *Mycoplasma hominis* dans le liquide amniotique au deuxième trimestre chez des patientes asymptomatiques et accouchement prématuré

Des études rétrospectives anciennes recherchant une association entre *Ureaplasma* spp. et *Mycoplasma hominis* détectés par une culture classique dans le liquide amniotique au deuxième trimestre de la grossesse et l'accouchement prématuré ont été publiées [23]. L'association avec l'accouchement prématuré n'était pas constante. Mais, la méthodologie utilisée était parfois douteuse avec un nombre de perdus de vue important et *Mycoplasma hominis* et *Ureaplasma* spp. n'étaient pas toujours recherchés à l'aide de milieux de culture adaptés. En effet, les mycoplasmes sont des organismes labiles sans paroi cellulaire, ce qui les rend difficiles à cultiver. Un autre moyen de les rechercher est la technique de PCR. Elle permet une limite de détection inférieure à 12.5 CFU pour *Ureaplasma* spp. comme pour *Mycoplasma hominis* [24] et améliore la détection des microorganismes cibles de plus de 20%, en comparaison des techniques de culture habituelles [25]. Par ailleurs la technique de PCR détecte les organismes morts.

Récemment ont été publiés trois travaux rétrospectifs montrant qu'une PCR positive pour *Ureaplasma* spp. ou *Mycoplasma hominis* dans le liquide amniotique prélevé par amniocentèse pour un diagnostic prénatal au deuxième trimestre étaient associées à l'accouchement prématuré [26].

La prévalence de ces germes dans le liquide amniotique était de l'ordre de 11 % pour *Ureaplasma* spp. et de 6 % pour *Mycoplasma hominis* entre 15 et 19 SA (tableau V). Gerber et coll ont recherché *Ureaplasma* spp. par PCR sur une population de 254 patientes prélevées par amniocentèse sans facteurs de risque d'accouchement prématuré. Vingt-quatre pour cent

des patientes PCR + ont accouché avant 37 SA versus 0.4% parmi les patientes PCR - (p<0.001) [27]. Le même type d'étude a été réalisé pour *Mycoplasma hominis* par Nguyen et coll. [28]. La prévalence de *Mycoplasma hominis* dans l'étude était de 6.4 % sur une population de 456 patientes. En cas de PCR +, 10,4 % des patientes accouchaient prématurément versus 1.9 % en cas de PCR - (p=0.02).

Ces travaux rétrospectifs récents montrent que *Mycoplasma hominis* et *Ureaplasma* spp. sont détectables par PCR dans le liquide amniotique au second trimestre de la grossesse. La présence de ces bactéries serait le témoin d'une invasion de la décidue, du chorion et de l'amnios qui aboutirait à la production de médiateurs pro-inflammatoires puis finalement à l'accouchement prématuré.

1.4 Utilisation d'une antibiothérapie par la Josamycine versus placebo pour la prévention de l'accouchement prématuré.

Plusieurs essais thérapeutiques utilisant des antibiotiques ont été réalisés ces 10 dernières années pour tenter de réduire le risque d'accouchement prématuré.

Les antibiotiques permettent de prolonger la grossesse lorsque le risque infectieux est très élevé comme en cas de rupture des membranes avant 37 SA [29]. En revanche dans des situations cliniques où le risque infectieux est moins important comme la menace d'accouchement prématuré à membranes intactes le bénéfice du traitement antibiotique n'a pas été démontré [30].

En cas de vaginose bactérienne (définie plus haut), l'antibiothérapie permet de réduire le risque d'accouchement prématuré, mais seulement chez les patientes qui ont un antécédent d'accouchement prématuré et en cas de traitement précoce au cours de la grossesse (tableau VII) [31].

Il n'existe pas d'essai prospectif qui ait testé un traitement antibiotique en utilisant comme critère de traitement une PCR positive pour *Ureaplasma* spp. et/ou *Mycoplasma hominis* dans le liquide amniotique. Pourtant une colonisation bactérienne du liquide amniotique détectée par PCR pourrait être un indicateur direct de colonisation de la décidue et des membranes foetales.

Un traitement antibiotique par la Josamycine peut réduire le risque d'accouchement prématuré dans certaines situations cliniques à haut risque infectieux. Une invasion bactérienne du liquide amniotique détectée par PCR pourrait être une cible de choix pour un traitement antibiotique mais il n'existe pas d'essai prospectif thérapeutique publié.

1.5 Intérêt d'un essai thérapeutique randomisé contre placebo

Des travaux récents ont montré que la détection par PCR de *Mycoplasma hominis* et *Ureaplasma* spp. dans le liquide amniotique au deuxième trimestre chez des patientes asymptomatiques est associée à une augmentation importante du risque d'accouchement prématuré [26-28]. Il paraît alors licite de rechercher et éventuellement de traiter en cas de PCR positive pour une de ces deux bactéries qui témoignerait d'une atteinte de la décidue et des membranes fœtales.

Néanmoins, ces études sont rétrospectives et ne permettent pas d'affirmer de relation causale avec l'accouchement prématuré même si elle semble probable. Par ailleurs le traitement peut être trop tardif pour prévenir la survenue d'un accouchement prématuré. Pour ces raisons, un essai multicentrique randomisé contre placebo est le meilleur moyen de démontrer l'efficacité d'un traitement antibiotique pour diminuer le risque d'accouchement prématuré en cas de PCR positive pour *Mycoplasma hominis* et/ou *Ureaplasma* spp. dans le liquide amniotique.

2 Objectif de l'étude

Tester l'efficacité d'une antibiothérapie par la Josamycine en cas de PCR positive pour *Ureaplasma* spp. et/ou *Mycoplasma hominis* pour réduire la prématurité spontanée chez les patientes asymptomatiques qui bénéficient d'une amniocentèse au deuxième trimestre.

3 Type d'étude

Il s'agit d'un essai thérapeutique dans le cadre d'une étude multicentrique randomisée contre placebo en double insu.

Sept maternités ayant un centre de diagnostic prénatal et un laboratoire de bactériologie participeront à l'étude.

Le monitorage et la randomisation seront assurés par l'URC du CHU Henri Mondor.

Le conditionnement du médicament et du placebo sera assuré par un le laboratoire Bayer. La numérotation des lots, l'expédition aux pharmacies centrales des centres investigateurs, les retours, les destructions et les études de stabilité du principe actif seront réalisés par l'unité d'essai clinique de l'AGEPS.

L'aspect méthodologique et statistique est assuré par l'Unité de Recherche épidémiologique sur la santé des femmes et des enfants (INSERM U 149).

4 Population étudiée

La population étudiée sera uniquement composée de patientes qui ont une amniocentèse au cours de la grossesse dans le but de réaliser un diagnostic prénatal. En effet **il n'y a aucun geste invasif réalisé pour l'étude** et aucun surcroît de risque lié au prélèvement pour la patiente. Seuls 3 ml de liquide amniotique seront réservés pour l'étude sur la quantité totale prélevée normalement chez la patiente qui est variable (entre 15 et 40 ml) en fonction des objectifs du diagnostic prénatal. Ces 3 ml supplémentaires n'ont aucune incidence sur le risque de perte fœtale secondaire à l'amniocentèse qui est attribuable à la ponction elle-même et non à la quantité prélevée.

Il est possible que la population étudiée soit différente de la population générale. En effet, les patientes qui bénéficient d'une amniocentèse sont plus âgées et présentent un risque de complications obstétricales au cours de la grossesse qui peut être différent des femmes plus jeunes en particulier en ce qui concerne l'hypertension artérielle et la prééclampsie. Néanmoins il n'y a pas de démonstration d'un surcroît de risque d'accouchement prématuré lié à l'âge.

Enfin, l'amniocentèse entraîne un surcroît de risque de perte fœtale évalué à 0.6 à 1% au cours de la grossesse [32], mais cela ne fausse pas la comparabilité entre les groupes puisque toutes les patientes ont une amniocentèse.

5 Critères d'inclusion

- Patiente majeure (≥ 18 ans)
- Parlant et comprenant le français
- Patiente affiliée à la sécurité sociale ou à un régime équivalent
- Ayant une indication d’amniocentèse pour analyse du caryotype et une échographie morphologique normale (en dehors de signes mineurs de trisomie 21)
- Avec à l’amniocentèse un liquide amniotique clair (non contaminé par le sang maternel)
- Dont l’âge gestationnel est compris entre 15 et 20 SA
- N’ayant pas d’allergie connue aux macrolides
- Patiente suivie pendant sa grossesse dans un centre investigateur
- Donnant son consentement éclairé et signé

6 Critères d'exclusion

- Patiente mineure
- Ne comprenant pas le français
- Ayant une allergie aux macrolides
- Ayant une grossesse multiple
- Anomalie morphologique à l’échographie (en dehors des signes mineurs de trisomie 21)
- Patiente refusant de participer à l’essai quelle qu’en soit la raison
- Intolérance connue au Lactose

7 Facteur étudié : PCR pour *Mycoplasma hominis* et *Ureaplasma* spp.

Pour que la procédure de réalisation des PCR soit homogène, elle sera totalement réalisée dans le laboratoire de bactériologie de Cochin. Cela a une implication directe sur les centres qu'il est possible d'inclure dans l'étude. La distance joue en effet un rôle majeur, un éloignement important des centres empêcherait un acheminement rapide indispensable pour l'étude. D'autre part cela nécessiterait des conditions de transport particulières (congélation à –80°C) qui rendraient les coûts très élevés.

La recherche de *Mycoplasma hominis* et d'*Ureaplasma* spp. (*U. parvum* et *U. urealyticum)* sera réalisée par PCR sur le liquide amniotique après extraction de l'ADN. Brièvement, l'ADN sera extrait à partir de 200 µl de chaque échantillon à l'aide du kit d'extraction Qiagen. Les PCR seront réalisées à l'aide d'amorces spécifiques de zones hautement conservées qui se trouvent dans le gène de l'uréase pour *Ureaplasma* spp. et dans le gène de l'ARN 16S pour M. hominis (tableau VII). Les séquences de ces amorces sont décrites, évaluées et publiées dans plusieurs études [24, 26-28].

La spécificité du produit d'amplification sera évaluée par une technique ELISA au cours de laquelle les amplicons seront dénaturés, biotinylés et évalués par mesure de la densité optique d'une réaction colorimétrique. Les produits amplifiés seront également caractérisés par leur taille, après migration sur gel d'agarose contenant du BET. Pour *Mycoplasma hominis* le fragment obtenu sera également soumis à une digestion enzymatique permettant une identification plus spécifique. La technique de PCR pourra également permettre la distinction entre les deux espèces d'*Ureaplasma* récemment identifiées (*U. parvum* et *U. urealyticum).*

8 Antibiotiques utilisés

Le site d'action désiré des antibiotiques est la décidue, le chorion et l'amnios. En effet une PCR positive dans le liquide amniotique témoigne essentiellement d'une atteinte de ces tissus choriodéciduaux et ceux ci doivent être préférentiellement concernés par l'antibiothérapie.

Les bactéries ciblées par l'antibiothérapie sont *Mycoplasma hominis* et *Ureaplasma* spp.

Les tétracyclines, les macrolides et apparentés (lincosamides dont la clindamycine) et les fluoroquinolones sont les principales familles d'antibiotiques actives contre ces bactéries [33].

Les antibiotiques utilisables sont les macrolides et les lincosamides car les tétracyclines et les fluoroquinolones sont contre-indiquées pendant la grossesse et chez l'enfant.

Ureaplasma spp. est sensible aux macrolides mais résiste aux lincosamides. A l'inverse, *Mycoplasma hominis* est résistant aux macrolides à 14 et 15 atomes de carbone comme l'érythromycine mais est sensible à certains macrolides à 16 atomes de carbone (josamycine et midécamycine) et aux lincosamides. Parmi les différents macrolides, la josamycine représente le meilleur compromis pour son activité sur les deux espèces bactériennes avec des concentrations minimales inhibitrices pour *Mycoplasma hominis* < 1mg/L et pour *Ureaplasma* spp. < 2mg/L (tableau VIII) [34].

Une posologie de deux grammes par jour de josamycine pendant 10 jours permettrait d'avoir des concentrations intratissulaires suffisantes pour être actives sur les mycoplasmes pendant une durée suffisamment longue pour diminuer le risque de récidive.

L'antibiotique utilisé en cas de PCR positive sera la Josamycine à la posologie de 1 gramme matin et soir par voie orale pendant 10 jours.

9 Effets indésirables de la Josamycine

Les effets secondaires décrits avec la josamycine sont :

Troubles gastro-intestinaux :
Nausées, vomissements, gastralgies, diarrhée, douleurs abdominales, colite pseudomembraneuse.
Troubles cutanés et sous-cutanés :
Éruption érythémateuse ou maculopapuleuse, érythème multiforme, dermatose bulleuse, syndrome de Stevens-Johnson, syndrome de Lyell.
Troubles du système immunitaire : Réactions d'hypersensibilité à type de prurit, urticaire, oedème de la face, oedème de Quincke (angioedème), gêne respiratoire, réaction et choc anaphylactique.Maladie sérique.
Affection hépato-biliaire :
Augmentation des transaminases et phosphatases alcalines, ictère, hépatite cholestatique ou hépatite cytolytique.
Troubles vasculaires :
Purpura, vascularite cutanée.

En cas d'événement indésirable grave, une procédure de déclaration spécifique sera réalisée (Annexe III remise en page).

10 Impact de la josamycine sur le risque de résistance de la flore vaginale bactérienne

La prescription d'une antibiothérapie peut sélectionner une flore bactérienne résistante aux antibiotiques.

Un prélèvement vaginal est habituellement réalisé entre 35 et 37 SA pour toutes les femmes enceintes suivant les recommandations de l'ANAES. L'objectif de ce prélèvement est de rechercher un streptocoque B pour que des mesures prophylactiques puissent être prévues à l'accouchement. Un antibiogramme sera également réalisé pour rechercher une résistance du streptocoque B aux macrolides. Ce contrôle ne sera réalisé que pour les patientes qui participeront à l'essai thérapeutique (josamycine ou placebo). Les résultats de ce prélèvement seront connus par le médecin ayant en charge la patiente.

11 Traitements concomitants

L’étude n’entraîne pas de modification de la prise en charge habituelle de la grossesse. Si un autre traitement antibiotique doit être prescrit, cela sera noté dans le CRF électronique.

Les associations médicamenteuses contre indiquées, déconseillées ou nécessitant des précautions d’emploi sont décrites ci-dessous. Ces médicaments ne sont que très rarement prescrits pendant la grossesse et constitueront une contre indication à l’inclusion dans l’étude ou un motif d’exclusion secondaire si ce traitement est prescrit secondairement:

Contre-indiquées :

- Dihydroergotamine, ergotamine : ergotisme, avec possibilité de nécrose des extrémités (diminution de l'élimination hépatique des alcaloïdes de l'ergot de seigle).
- Cisapride : risque majoré de troubles du rythme ventriculaire, notamment de torsades de pointes.
- Pimozide : risque majoré de troubles du rythme ventriculaire, notamment de torsades de pointes.

Déconseillées :

- Ébastine : risque majoré de troubles du rythme ventriculaire, notamment chez les sujets prédisposés (syndrome du QT long, congénital).
- Alcaloïdes de l'ergot de seigle dopaminergiques (bromocriptine, cabergoline, lisuride, pergolide) : augmentation des concentrations plasmatiques du dopaminergique avec accroissement possible de son activité ou apparition de signes de surdosage.
- Triazolam : quelques cas de majoration des effets indésirables (troubles du comportement) du triazolam ont été rapportés.
- Halofantrine : risque majoré de troubles du rythme ventriculaire, notamment de torsades de pointes. Si cela est possible, interrompre le macrolide. Si l'association ne peut être évitée, contrôle préalable du QT et surveillance ECG monitorée.
- Disopyramide : risque de majoration des effets indésirables du disopyramide : hypoglycémies sévères, allongement de l'intervalle QT et troubles du rythme ventriculaire graves, notamment à type de torsades de pointes. Surveillance clinique, biologique et électrocardiographique régulière.
- Colchicine : augmentation des effets indésirables de la colchicine aux conséquences potentiellement fatales.

Nécessitant des précautions d'emploi :

- Carbamazépine : augmentation des concentrations plasmatiques de carbamazépine avec signes de surdosage par inhibition de son métabolisme hépatique. Surveillance

clinique et, si besoin, dosage plasmatique et réduction éventuelle de la posologie de la carbamazépine.

- Ciclosporine : risque d'augmentation des concentrations sanguines de ciclosporine et de la créatininémie. Dosage des concentrations sanguines de la ciclosporine, contrôle de la fonction rénale et adaptation de sa posologie pendant l'association et après l'arrêt de la josamycine.
- Anticoagulants oraux : augmentation de l'effet de l'anticoagulant oral et du risque hémorragique. Contrôle plus fréquent de l'INR. Adaptation éventuelle de la posologie de l'anticoagulant oral pendant le traitement par la josamycine et après son arrêt.

12 Critères de jugement

12.1 Critère de jugement principal

Survenue d'un accouchement prématuré entre 22 et 37 SA

12.2 Critères de jugement secondaires.

12.2.1 Obstétricaux

Prénataux :

- Survenue d'une fausse couche tardive entre 16 et 22 SA
- Accouchement prématuré ≤ 34, 32, 28 SA.
- Hospitalisation pour menace d'accouchement prématuré
- Nombre de jours d'hospitalisation pour menace d'accouchement prématuré
- Rupture prématurée des membranes avant 37 SA
- Survenue d'une chorioamniotite définie par deux des critères suivants : une température maternelle > 38°C, des contractions utérines, des leucorrhées fétides, une tachycardie fœtale>160 bpm, une CRP>10 mg/L.

Pendant le travail :

- Hyperthermie > 38°C
- Tachycardie fœtale > 160 bpm

Dans le post-partum :

- Hyperthermie > 38°C durant plus de 24 heures
- Nécessité d’un traitement antibiotique pendant plus de 48 heures

12.2.2 Néonataux

12.2.2.1 Mortalité néonatale

- Mortalité néonatale précoce de J0 à J6
- Mortalité néonatale tardive de J7 à J28.

12.2.2.2 Morbidité néonatale

Etat néonatal immédiat

L'état néonatal immédiat sera jugé sur le score d'Apgar, le pH au cordon et le transfert en réanimation néonatale ou en unité de soins intensifs.

L'infection néonatale :

Elle est le plus souvent due à des streptocoques du groupe B ou à *Escherichia coli* et pourrait augmenter le risque de leucomalacie ventriculaire et de dysplasie bronchopulmonaire lorsqu'elle succède à une inflammation *in utero* [35]. L'expression clinique d'une infection chez le nouveau-né est polymorphe. Toute anomalie de l'examen clinique ne s'intégrant pas à l'évidence dans une autre pathologie sera considérée comme un signe d'infection : détresse respiratoire non liée à un trouble de résorption ou à une maladie des membranes hyalines, troubles hémodynamiques, anomalie de l'examen neurologique.

Les paramètres biologiques étudiés seront, les cultures périphériques, les cultures centrales, la CRP (positive si supérieure ou égale à 10 mg/l), la leucocytose (positive si supérieure à 25000 ou inférieure à 5000).Ces résultats sont issus d'examens réalisés systématiquement chez le nouveau né. Aucun examen supplémentaire ne sera réalisé pour cette recherche sur les nouveaux nés.

On considérera comme :

- Une infection certaine : Des prélèvements périphériques ou centraux positifs et une élévation de la CRP supérieure à 10 mg/l
- Une infection probable: Un examen clinique anormal attribuable à une infection ou un contexte de chorioamniotite maternelle et une élévation de la CRP supérieure à 10 mg/l.

L'absence des critères énumérés ci-dessus fera considérer que le nouveau-né n'est pas infecté.

Morbidité respiratoire :

- Immédiate :
  - Détresse respiratoire transitoire
  - Maladie des membranes hyalines
- Tardive :
  - Oxygénodépendance à 28 jours
  - Oxygénodépendance à 36 SA

Morbidité cérébrale

- Hémorragie intra ventriculaire grade 1-2-3-4 selon les critères de Papile [36]
- Leucomalacie périventriculaire

Morbidité digestive

- Survenue d'une entérocolite ulcéronécrosante selon la classification de Bell [37].

13 Réalisation pratique de l'étude (annexe I)

13.1 Inclusion des patientes et remplissage de la fiche d'inclusion : rôle de l'investigateur et du technicien de recherche clinique

La participation à la recherche sera proposée aux patientes qui se présenteront dans les services investigateurs pour la réalisation d’une amniocentèse entre 15 et 20 SA pour un diagnostic prénatal. Tous les critères seront vérifiés par le médecin investigateur du centre.

Il sera proposé aux patientes remplissant tous les critères d’inclusion de participer à l’essai PREMYC.

Le déroulement de l’étude sera expliqué à la patiente (notice d’information). Le médecin chargé de l’amniocentèse recueillera un volet du consentement éclairé signé par lui-même et par la patiente. Il sera précisé à la patiente qu’elle peut refuser de participer à l’essai, ou retirer son consentement en cours d’étude, sans que sa prise en charge et ses relations avec son médecin en soient affectées.

Lors de l’inclusion, le médecin remplira la fiche d’inclusion de la patiente sur un CRF électronique sécurisé (logiciel « cleanweb »).

Cette saisie initiale signalera l’inclusion de la patiente au technicien de recherche clinique qui assurera ensuite de la saisie des données concernant la patiente au cours de l’étude.

L’utilisation de ce CRF électronique informatisé directement accessible par Internet facilitera l’interaction entre les centres cliniques, le laboratoire de bactériologie, l’URC d’Henri Mondor et le DRCD. Par ailleurs, une grande partie de la saisie pourra être faite de manière centralisée par le technicien de recherche clinique. Enfin une grande partie du monitorage par l’attaché de recherche clinique pourra être réalisée de manière centralisée.

13.2 Anonymisation

Les données de l'étude seront anonymisées, chaque patiente étant repérée par :

- la première lettre de son nom de jeune fille
- la première lettre de son prénom
- le numéro du centre
- le numéro d'inclusion

Ces paramètres d'identification figureront sur les prélèvements et le eCRF.

13.3 Envoi du prélèvement au laboratoire de bactériologie

Les échantillons de liquide amniotique destinés à la recherche (PCR pour Mycoplasma hominis et Ureaplasma spp) seront conservés à 4°C dans un milieu spécifique pour une durée maximale de 3 jours et acheminés par le coursier de l'APHP au laboratoire de bactériologie de l'hôpital Cochin où ils seront réceptionnés par le technicien de laboratoire.

13.4 Sélection des patientes pour l'essai thérapeutique

Le résultat de la PCR sera transmis le jour même par télécopie au médecin investigateur (et à l'URC Henri Mondor).

Si la PCR est négative, la patiente sera informée du résultat par téléphone. Le médecin lui annoncera qu'elle ne participera pas à l'essai thérapeutique mais que le suivi de sa grossesse sera effectué de manière classique et que les données recueillies pendant ce suivi seront utilisées pour les besoins de la recherche (jusqu'à l'accouchement et sa sortie de maternité). La patiente sera revue par le médecin investigateur lors des visites programmées

dans le cadre du suivi normal de sa grossesse. Aucune visite n'étant effectuée spécifiquement pour la recherche, celle-ci n'entraîne donc aucun frais supplémentaire pour la patiente.

Si la PCR est positive pour *Ureaplasma* spp. et/ou *Mycoplasma hominis*, la patiente sera contactée par le technicien de recherche clinique qui lui proposera un rendez vous avec le médecin pour lui annoncer ce résultat et lui proposer le lot de traitement.

Le médecin s'assurera auprès de la patiente de sa volonté de poursuivre la recherche et de participer à l'essai thérapeutique.

Si la patiente ne souhaite plus participer à l'essai thérapeutique, la patiente sera sortie de l'étude. Le suivi de sa grossesse sera assuré selon les pratiques courantes du service, et dans le meilleur intérêt pour la patiente et son futur enfant.

Si la patiente souhaite continuer sa participation, un rendez-vous lui sera proposé pour une visite de dispensation du traitement (si contact téléphonique). Lors de cette visite, la patiente sera randomisée dans le groupe (Josamycine) ou dans le groupe (Placebo). Ni les médecins, ni les patientes ne sauront dans quel groupe elles appartiennent. Les frais de déplacement des patientes seront pris en charge par le promoteur de la recherche.

13.5 Randomisation et Administration des traitements

La randomisation s'effectuera selon une liste de randomisation stratifiée sur les centres, préparée par l'URC Henri Mondor.

La randomisation sera directement réalisée en ligne à partir du logiciel cleanweb.

Le lot de traitement sera alors remis à la patiente par la pharmacie, sur ordonnance spécifique de l'investigateur (Josamycine ou placebo, à la posologie de 1 gramme matin et soir par voie orale pendant 10 jours).

Une carte de levé d'insu sera remise aux patientes. (Voir chapitre sur la levée d'insu)

Les lots de traitements seront répartis dans les pharmacies des centres investigateurs par l'AGEPS. Ils seront étiquetés conformément à la législation en rigueur pour les médicaments destinés aux essais thérapeutiques. Afin de garantir l'insu, les lots de traitement de josamycine ou de placebo seront d'aspect identique

13.6 Contrôle de l'observance du traitement

Une procédure de suivi et de rappel des patientes sera réalisée pour le contrôle de l'observance au traitement. Un rappel téléphonique des patientes aura lieu 15 jours après le début du traitement et contrôlera à l'aide d'un questionnaire court l'observance et l'absence d'effets secondaires liés au traitement (annexe II)

Il sera également demandé à la patiente de retourner le coffret de traitement vierge, entamé ou vide lors de sa prochaine consultation. Les visites de consultations se feront mensuellement jusqu'à l'accouchement. Ces coffrets seront conservés dans les pharmacies des centres jusqu'à la fin de la recherche.

13.7 Signalement des patientes incluses dans le dossier médical

L'inclusion de la patiente dans l'étude sera repérée par une pastille collée sur la page de garde de son dossier clinique. . Si la patiente est incluse et randomisée une deuxième étiquette sera apposée sur le dossier médical afin de signaler qu'elle prend soit un antibiotique soit un placebo.

13.8 Suivi des patientes

Le suivi de toutes les patientes PCR+/PCR- sera consigné sous la responsabilité de l'investigateur par le technicien de recherche clinique.

Afin de contrôler d'éventuels facteurs de confusion, des données concernant la prise en charge, l'évolution de la grossesse, de l'accouchement ainsi que les caractéristiques à la naissance du nouveau-né seront recueillies.

Le suivi maternel sera réalisé jusqu'à la sortie de la maternité.

Le suivi des nouveau-nés prématurés sera effectué jusqu'à un âge corrigé de 36 semaines.

Le suivi des nouveau-nés à terme sera réalisé jusqu'à la sortie de la maternité.

Des données concernant l'évolution de la grossesse après l'inclusion dans l'étude seront recueillies.

Les données concernant le prélèvement vaginal entre 35 et 37 SA seront recueillies.

Les données concernant la prescription d'antibiotiques pendant la grossesse seront recueillies.

Les données concernant l'accouchement et le suivi néonatal avant la sortie de la maternité seront recueillies.

13.9 Recueil des données

Les données seront recueillies au fur et à mesure de l'avancée de l'étude directement sur ordinateur dans le CRF électronique à l'aide du logiciel « cleanweb » .

Les données seront recueillies directement par le technicien de recherche clinique au fur et à mesure de l'accouchement des patientes.

14 Levée de l'insu

En cas de nécessité, la levée d'insu sera réalisée suivant la procédure du Centre Fernand Widal. Les patientes participant à l'essai thérapeutique porteront sur elles une carte patient sur laquelle seront indiqués les numéros de téléphone du Centre Fernand Widal et de l'investigateur (Cf modèle de carte).

Nom :................................<br>Prénom : ....<br>**Je participe à la recherche biomédicale : PREMYC**<br><br>Je reçois le traitement suivant : **JOSAMYCINE 2g/jour ou Placebo**<br><br>N°Traitement : ....<br><br>Je suis suivi par le Dr ……………………………………N° Téléphone………..…<br>A l'hôpital …………………………………………………<br><br>En cas d'urgence, vous pouvez contacter le médecin de garde du Centre Anti-Poison Hôpital Fernand Widal 24h/24, 7j/7 au : ☎ 01 40 05 48 48

Tout effet indésirable grave fera l'objet d'une déclaration spécifique (Annexe III et IV).

15 Conditionnement et distribution de l’antibiotique et du placebo

Le conditionnement du médicament et du placebo sera assuré par Bayer. La numérotation des lots, l’expédition aux pharmacies centrales des centres investigateurs, les retours, les destructions et les études de stabilité du principe actif seront réalisés par l’unité d’essai clinique de l’AGEPS.

16 Statistiques

16.1 Nombre de sujets nécessaires

Nous avons estimé le risque d'accouchement prématuré à 17 % en cas de PCR positive du liquide amniotique pour *Ureaplasma* spp. ou *Mycoplasma hominis,* (hypothèse moyenne entre les 10 à 24 % correspondant aux chiffres publiés). Pour pouvoir montrer une réduction du risque d'accouchement prématuré de 50 % il est nécessaire d'inclure 238 femmes dans chaque groupe (α=0.05, 1-ß = 0.80).

Les prévalences publiées au deuxième trimestre de PCR positive pour *Ureaplasma* spp. et *Mycoplasma hominis* varient peu en fonction des études et des pays (Etats-Unis ou Suisse) et sont de 15 % au minimum en cumulant les deux bactéries [26]. L'inclusion de 3200 patientes qui bénéficient d'une amniocentèse permettra d'atteindre le nombre voulu de patientes.

16.2 Durée de l'étude et faisabilité

Le nombre d'amniocentèses réalisées sur une année sur l'ensemble des centres qui participent à l'étude est d'environ **3750 amniocentèses par an réparties ainsi**:

- CHI de Créteil : 400
- Port Royal : 500
- Saint Vincent de Paul : 550
- CHI de Poissy 650
- Rothschild Trousseau : 200
- Saint Antoine : 650
- Necker : 200
- Robert Debré : 600

En supposant un taux d'inclusion dans l'étude de 40 % (refus des patientes, grossesses gémellaires, malformations…), le nombre de 3200 patientes pourrait être atteint en 30 mois sur l'ensemble des centres ayant accepté de participer à l'étude. Le suivi des patientes et des nouveau-nés est de 6 mois après la date d'inclusion. La durée totale de l'étude serait donc de 36 mois. Le nombre de patientes perdues de vue devrait en principe être très faible car il s'agit de patientes enceintes qui bénéficient d'un suivi très régulier et pour lesquelles l'accouchement aura nécessairement lieu dans une maternité.

16.3 Analyse des données

La saisie des données sera réalisée au fur et à mesure sous la responsabilité de l'investigateur grâce au logiciel « cleanweb ». Le dossier informatique sera traité sous STATA (College Station, TX, USA)

L'analyse des données se fera dans l'unité INSERM U149 par F Maillard et G Kayem.

L'étude distinguera 3 groupes de patientes en fonction du facteur étudié et de la prescription d'un traitement antibiotique : avec une PCR négative, avec une PCR positive sans traitement antibiotique, avec une PCR positive avec traitement antibiotique.

Ces groupes seront comparés entre eux pour

- Les critères cliniques recueillis à l'inclusion : âge maternel et niveau d'études, antécédents obstétricaux, âge gestationnel à l'inclusion.
- Les critères recueillis en cours de grossesse : nombre de consultations, prise du traitement, traitements concomitants, sorties de protocole, évènements indésirables et levée d'insu.

Puis on recherchera en intention de traiter l'existence d'un effet « antibiotique » sous la forme d'une différence, en cas de PCR positive, entre le groupe « placebo » et le groupe « josamycine » pour le critère de jugement principal et les critères de jugements secondaires.

Les comparaisons feront appel à des tests statistiques non paramétriques à chaque fois que des contraintes concernant les effectifs ou les distributions le nécessiteront. Pour les variables catégorielles, on fera appel au test du chi2 et au test Fisher. On fera appel à l ‘analyse de variance et au test non paramétrique de Kruskal et Wallis pour les variables continues.

Les effets seront mesurés sous forme de risque relatif, avec leur intervalle de confiance à 95%.

En ce qui concerne les patientes perdues de vue, leurs caractéristiques seront étudiées. Une analyse de sensibilité sera réalisée pour savoir dans quelle mesure les résultats de l'étude auront pu être influencés par ce manque d'information.

17 Bénéfices attendus de l'étude

L'efficacité d'une antibiothérapie permettra de réduire la prématurité spontanée d'origine infectieuse chez les patientes qui subissent une amniocentèse. La prématurité spontanée attendue pour ces 80000 patientes est de 4 % soit 3200 patientes. En supposant que 320 à 960 (10 à 30 %) soient liés à une invasion microbienne précoce du liquide amniotique, une efficacité de l'antibiotique dans 50 % des cas permettrait de prévenir 160 à 480 accouchements prématurés par an en France.

18 Aspects réglementaires et législatifs

18.1 Déclaration CNIL

La loi prévoit que la déclaration doit avoir été faite avant le début effectif de la recherche.
Le DRCD en qualité de promoteur effectuera une déclaration à la CNIL, en relation avec le responsable du fichier informatique, lors de sa déclaration annuelle simplifiée si la recherche fait l'objet d'un contrôle qualité des données par un ARC et entre dans le champ d'application de la procédure simplifiée CNIL.
En sont exclues les recherches en génétique identifiante, épidémiologie ou l'étude des comportements, les recherches qui comportent des données sensibles en termes de confidentialité (identité complète des personnes ou numéro de sécurité sociale collecté). Dans ce cas, ainsi que pour les recherches non monitorées, le responsable du fichier assure lui-même la déclaration unitaire de la recherche auprès du Comité consultatif sur le traitement de l'information en matière de recherche dans le domaine de la santé puis de la CNIL.

18.2 Documentations de la recherche

Avant de démarrer la recherche, l'investigateur coordonnateur fournira au représentant du promoteur de la recherche une copie de son curriculum vitæ personnel daté et signé et comportant son numéro d'inscription à l'ordre des médecins, de même que tous les investigateurs.
La version du protocole acceptée avant soumission avec ses annexes sera signée conjointement par l'investigateur coordonnateur et le représentant du promoteur. Le cas échéant, le responsable scientifique sera également signataire.

Lors de chaque nouvelle version du protocole, rendue nécessaire par des amendements et/ou demandes des autorités, un nouveau numéro et la date seront attribués et les mêmes signatures recueillies.

Chaque investigateur s'engagera à respecter les obligations de la loi et à mener la recherche selon les B.P.C. et en respectant les termes de la déclaration d'Helsinki. Pour ce faire, un exemplaire daté et signé de l'engagement scientifique (document type DRCD) par chaque investigateur de chaque service clinique participant d'un centre sera remis au promoteur.

18.3 Contrôle de Qualité et Assurance Qualité

La recherche sera encadrée selon les procédures opératoires standard de l'AP-HP promoteur.
Le déroulement de la recherche dans les centres investigateurs et la prise en charge des sujets sera faite conformément à la déclaration d'Helsinki et les Bonnes Pratiques.
Procédures de monitoring
Les représentants du promoteur effectueront des visites des centres investigateurs au rythme correspondant au schéma de suivi des patients dans le protocole, aux inclusions dans les différents centres et au niveau de risque qui a été attribué au protocole.
D'abord, avant inclusions, pour une ouverture de chaque centre avec mise en place du protocole et prise de connaissance avec les investigateurs.
Lors des visites suivantes, les cahiers d'observation seront revus au fur et à mesure de l'état d'avancement de la recherche par les ARCs représentant le promoteur qui en contrôleront le bon remplissage et assureront la validation des données. L'investigateur principal de chaque centre et les autres investigateurs qui incluent ou suivent des sujets participant à la recherche acceptent de recevoir des représentants du promoteur nommés par l'AP-HP à intervalles réguliers.
Lors de ces visites sur site et en accord avec les Bonnes Pratiques Cliniques, les éléments suivants seront revus :
- respect du protocole de la recherche et des procédures qui y sont définies,
- examen des documents source et confrontation avec les données reportées dans le CRF électronique,
- assurance de qualité des données recueillies dans le CRF électronique : exactitude, données manquantes, cohérence des données, selon les règles édictées par les procédures de Le DRCD,

18.4 Gestion des Evénements Indésirables

18.4.1 Définitions :

Evénement indésirable

Toute manifestation nocive et non recherchée survenant chez une personne pendant une recherche, qu'elle soit considérée ou non comme liée à celle-ci.

Effet indésirable

Réaction nocive et non voulue à un traitement expérimental : médicament (quelle que soit la dose administrée), dispositif, traitement chirurgical…) utilisé chez l'homme,.

Effet ou événement indésirable grave

Effet ou événement indésirable ayant entraîné :

- Le décès
- La mise en jeu du pronostic vital
- Une invalidité ou une incapacité importantes ou durables
- Une hospitalisation ou prolongation d'hospitalisation
- Une anomalie ou malformation congénitale
- Autre : tout effet indésirable jugé comme grave par le professionnel de santé, en particulier les événements nécessitant une intervention pour éviter l'une des conséquences notées ci-dessus, et certains résultats d'examens para cliniques.

Effet indésirable inattendu

Effet indésirable dont la nature, la gravité ou l'évolution ne correspondent pas aux informations contenues dans le résumé des caractéristiques du produit, la brochure investigateur ou autre référentiel reconnu par les autorités.

18.4.2 Obligations des investigateurs :

Evénements indésirables non graves :

Tout événement indésirable - non grave suivant la définition précédente - observé lors de la recherche et dans ses suites devra être reporté dans le CRF électronique dans la section prévue à cet effet.

Un seul événement doit être reporté par item. L'événement peut correspondre à un symptôme, un diagnostic ou à un résultat d'examen complémentaire jugé significatif. Tous les éléments cliniques ou para cliniques permettant de décrire au mieux l'événement correspondant doivent être reportés.
Tout patient présentant un événement indésirable doit être suivi jusqu'à la résolution ou la stabilisation de celui-ci, et l'évolution en sera notée sur la page correspondante.

Evénements indésirables graves :
Les investigateurs doivent informer, en temps réel, l'AP-HP-promoteur d'éventuels événements indésirables graves tels que définis ci-dessus.
L'investigateur envoie les copies des feuillets-type événements indésirables graves du CRF électronique de la recherche, décrivant l'EIG, par fax, à Le DRCD au nom du chef de projet en charge de la recherche au 01 44 84 17 99 dans les 48 heures, (après si possible un appel téléphonique immédiat au 01 44 84 17 23 en cas de décès ou d'une menace vitale inattendus).
L'investigateur devra pour chaque événement indésirable grave émettre un avis médical sur la relation pouvant exister entre l'apparition de l'évènement et le protocole.
Les bilans cliniques, les examens diagnostiques et les examens de laboratoire appropriés seront mis en route afin d'identifier l'origine de la réaction et les résultats de ces explorations ainsi que l'évolution clinique seront rapportés.
Tout fait nouveau survenu dans la recherche ou dans le contexte de la recherche provenant de données de la littérature ou de recherches en cours doit aussi être notifié au promoteur très rapidement.

18.4.3 Déclaration des événements indésirables graves aux Autorités de Santé :

Elle sera assurée par le pôle de pharmacovigilance de Le DRCD, après analyse d'imputabilité de chaque cas. Toutes les suspicions d'effet indésirable grave inattendu (non présent dans le RCP ou la brochure investigateur du ou des produits utilisés dans la recherche) seront déclarées par le promoteur à l'autorité compétente dans les délais légaux.
En cas d'effet indésirable grave inattendu dû à l'un des traitements de la recherche ou la recherche elle - même, le CPP et les investigateurs de la recherche devront être informés.

18.5 Transcription des données dans le CRF électronique

Toutes les informations requises par le protocole doivent être fournies dans le CRF électronique et une explication donnée par l'investigateur pour chaque donnée manquante.

Les données devront être transférées dans les cahiers d'observation au fur et à mesure qu'elles sont obtenues qu'il s'agisse de données cliniques ou para-cliniques Les données devront être copiées de façon nette et lisible à l'encre noire dans ces cahiers (ceci afin de faciliter la duplication et la saisie informatique).

Les données erronées dépistées sur les cahiers d'observation seront clairement barrées et les nouvelles données seront copiées sur le cahier avec les initiales et la date par le membre de l'équipe de l'investigateur qui aura fait la correction.

L'anonymat des sujets sera assuré par la mention au maximum des 3 premières lettres du nom et des 2 premières lettres du prénom du sujet sur tous les documents nécessaires à la recherche, ou par effacement par les moyens appropriés (blanc correcteur…) des données nominatives sur les copies des documents source, destinés à la documentation de la recherche.

Les données informatisées sur un fichier seront déclarées à la CNIL selon la procédure adaptée au cas.

18.6 Amendements au protocole de la recherche

Le DRCD doit être informée de tout projet de modification du protocole par l'investigateur coordonnateur. Les modifications devront être qualifiées en substantielles ou non.

Tout amendement au protocole de la recherche, devra être notifié au CPP s'il entraîne des modifications substantielles, c'est-à-dire si les modifications prévues sont susceptibles, d'une manière ou d'une autre, de modifier les garanties apportées aux personnes qui se prêtent à la recherche biomédicale (modification d'un critère d'inclusion, prolongation d'une durée d'inclusion, participation de nouveaux investigateurs..…).

18.7 Extension de la recherche

Toute extension de la recherche (modification profonde du schéma thérapeutique ou des populations incluses, prolongation des traitements et ou des actes thérapeutiques non prévus initialement dans le protocole) devra être considérée comme une nouvelle recherche.

18.8 Responsabilité

L'Assistance Publique-Hôpitaux de Paris est le promoteur de cette recherche. En accord avec la loi sur les recherches biomédicales, elle a pris une assurance auprès de la compagnie

GERLING France pour toute la durée de la recherche, garantissant sa propre responsabilité civile ainsi que celle de tout intervenant (médecin ou personnel impliqué dans la réalisation de la recherche) (loi n°2004-806, Art L.1121-10 du CSP).
l'Assistance Publique - Hôpitaux de Paris se réserve le droit d'interrompre la recherche à tout moment pour des raisons médicales ou administratives; dans cette éventualité, une notification sera fournie à l'investigateur.

18.9 Rapport final de la recherche

Le rapport final de la recherche sera écrit en collaboration par le coordonnateur et le biostatisticien pour cette recherche. Ce rapport sera soumis à chacun des investigateurs pour avis. Une fois qu'un consensus aura été obtenu, la version finale devra être avalisée par la signature de chacun des investigateurs et adressée au promoteur dans les meilleurs délais après la fin effective de la recherche. Un rapport rédigé selon le plan de référence de l’autorité compétente doit être transmis à l’autorité compétente ainsi qu’au Comité dans un délai de un an, après la fin de la recherche, s’entendant comme la dernière visite de suivi du dernier sujet inclus. Ce délai est rapporté à 90 jours en cas d’arrêt prématuré de la recherche.

18.10 Publications et propriétés des données

L’AP-HP est propriétaire des données et aucune utilisation ou transmission à un tiers ne peut être effectuée sans son accord préalable.
Seront premiers signataires des publications, les personnes ayant réellement participé à l’élaboration du protocole et son déroulement ainsi qu’à la rédaction des résultats.
L’Assistance Publique- Hôpitaux de Paris doit être mentionnée comme étant le promoteur de la recherche biomédicale et comme soutien financier le cas échéant. les termes « Assistance Publique- Hôpitaux de Paris » doivent apparaître dans l’adresse des auteurs.

18.11 Formulaire d’information aux sujets et consentement

Les patientes ne pourront participer à cette étude que si elles ont donné leur consentement par écrit. L'inclusion définitive n'interviendra qu'après obtention de ce consentement.

Les patientes recevront une information de la part de leur médecin sur : le but de cette étude, la durée de leur participation, les procédures qui seront suivies, les contraintes, les risques, bénéfices, la confidentialité des données, la couverture par une assurance. L'ensemble de ces informations est résumé sur une note d'information remise à chaque patiente qui sera datée et signée en trois exemplaires par la patiente et le médecin investigateur. Une copie de ce document

sera remise à la personne participant à l'étude ; l'investigateur devra garder le deuxième exemplaire dans ses archives pendant un minimum de 15 ans ; le troisième exemplaire sera remis à la fin de l'étude au promoteur sous enveloppe scellée.

18.12 Soumission au CPP et déclaration à l'AFSSAPS

Le promoteur de l'étude soumettra ce protocole simultanément au Comité de Protection des Personnes et à l'AFSSAPS en la loi n° 2004-806 du 9 août 2004. Le promoteur contractera une assurance correspondant aux risques éventuellement encourus par les patientes durant l'étude du fait de leur participation. Cette assurance couvre les intervenants dans la recherche (investigateurs, responsabilité civile)

18.13 Amendement au protocole de l'étude et extension de l'étude

Tout amendement ou extension de l'étude seront notifiés après accord du promoteur au CPP et à l'AFSSAPS.

18.14 Déclaration CNIL

Conformément à la réglementation l'étude fera l'objet d'une procédure de déclaration à la CNIL.

18.15 Documentation de l'étude

Avant de commencer l'étude, les investigateurs fourniront aux représentants du promoteur une copie de leur curriculum vitae personnel. Ils s'engageront à respecter les obligations de la loi n° 2004-806 du 9 août 2004, les termes de la déclaration d'Helsinki, à mener cette étude selon les Bonnes Pratiques Cliniques (BPC) et selon le protocole. Un exemplaire daté et signé de l'engagement scientifique sera remis aux représentants du promoteur.

Tableau I. Morbidité associée à une culture positive du liquide amniotique en cas de menace d'accouchement prématuré à membranes intactes.

Auteur	N	Cultures + (%)	Myc	Chorioamniotite (%) *	AP (%)	OR* (IC95%)
Gravett [38]	54	13(24)	Oui	5(38.5)	5(38,5)	
Romero [39]	41	4(9.8)	Oui			
Romero [40]	264	24(9.1)	Oui	3(12.5)	24(100)	2.75 (2.3-3.2)
Romero [41]	109	15(13.8)	Oui		15 (100)	
Romero [42]	168	23 (13.6)	Oui	4 (17.4)		
Gauthier [43]	113	18 (15.9)	Oui			
Romero [44]	195	25 (12.8)	Oui	4(16)	25 (100)	1.97 (1.6-2-3)
Coultrip[45]	107	12 (11.2)	Oui	7(58.3)		
Watts[46]	105	20 (19)	Oui		17(85)	
Romero [47]	120	11 (9.2)	Oui	2 (18.2)	11(100)	
Coultrip [48]	89	12 (13.5)	Oui	7(58.3)		
Yoon [49]	102	11(10.8)	Oui	2(18.3)		
Markenson [50]	54	5 (9.3)	Oui		5(100)	2.64(0.3-20.9)
Oyarzun [51]	50	6 (12)	Oui		4(66.7)	1.3 (0.-6.6)

*AP : accouchement prématuré, Myc : recherche de mycoplasmes en utilisant les milieux de culture appropriés, * : accouchement prématuré chez les patientes ayant une culture positive*

Tableau II. Morbidité associée à une culture positive du liquide amniotique en cas de menace d'accouchement prématuré avec rupture des membranes.

Auteur	N	Culture (+) %	Myc	Chorioamniotite *	INN *
Romero [52]	230	65/221(29.4)	Oui		5(12.8)
Coultrip[45]	29	12/29(41.4)	Oui	3(25)	
Romero [53]	110	42/110(38.2)	Oui	5(11.9)	20(47.6)
Averbuch[54]	90	32/90(35.6)	Oui		7 (21.9)
Yoon [22]	154	37/154(24)	Oui	7(18.9)	10/32(31.2)

*INN :infection néonatale. * : chez les patientes ayant une culture positive . Myc : recherche de mycoplasmes en utilisant les milieux de culture appropriés.*

Tableau III. Mycoplasmes colonisant le tractus urogénital dans l'espèce humaine.

Bactéries	Site de colonisation		Rôle pathogène
	Tractus respiratoire	Tractus urogénital	
Mycoplasma fermentans	+	+	oui?
Mycoplasma genitalium	–	+	oui
Mycoplasma hominis	–	+	oui
Mycoplasma penetrans	–	+	?
Mycoplasma primatum	+	+	non
Mycoplasma spermatophilum	–	+	non
Ureaplasma parvum	–	+	oui
Ureaplasma spp.	–	+	oui

A partir de [19]

Tableau IV. Pathologies associées ou causées par *Mycoplasma hominis* et *Ureaplasma* spp. chez la femme.

Pathologie	*Ureaplasma* spp.	*M. hominis*
Vaginose bactérienne	±	±
Cervicite	–	–
Inflammation pelvienne	–	+
Infertilité	±	–
Chorioamnionite	+	±
Avortement spontané	+	±
Prématurité	+	±
Retard de croissance intra utérin	±	–
Endométrite	+	+
Pathologie extragénitale (arthrite)	+	+

–, pas d'association; ±, association significative; +, rôle causal probable

A partir de [19, 27, 28]

Tableau V. PCR positive pour *Ureaplasma* spp. et/ou *Mycoplasma hominis* au second trimestre de la grossesse chez des patientes asymptomatiques et risque d'accouchement prématuré.

	année	n	Bactéries recherchées	Prévalence N (%)	AP < 37 SA si PCR+ (%)	AP (OR, IC 95%)
Gerber [27]	2003	254	*Ureaplasma* spp.	29 (11,4)	24.1	54.3 (6.5-457.3)
Perni [26]	2004	172	*Mycoplasma hominis*, *Ureaplasma* spp.	32(17.9)	15.6	12.9 (2.4 – 69.6)
Nguyen[28]	2004	456	*Mycoplasma hominis*	29 (6.4)	10.7	4.7 (1.4-15.4)

AP : accouchement prématuré

Tableau VI. Essais randomisés de prévention de l'accouchement prématuré d'origine infectieuse par le traitement d'une colonisation vaginale anormale à l'aide d'un traitement antibiotique par voie orale.

	Année	N	Population étudiée	Bactéries recherchées	Antibiotique utilisé	AG inclusion (SA)	accouchement prématuré (traité vs pas de traitement)	p
Kiss [55]	2004	4155	population générale	Vaginose bactérienne *Candida* *Trichomonas vaginalis*	clindamycine crème et si échec per os clotrimoxazole métronidazole	15-20	3% vs 5.3% [a]	<0.001
Ugwumadu [31]	2003	485	uniquement si vaginose bactérienne	Vaginose bactérienne	clindamycine 300 mg/j 5 j	15-16	5% vs 12%	0.001
Klebanoff [11]	2001	617	uniquement si Trichomonas vaginalis	*Trichomonas vaginalis*	métronidazole 2 g	8-23	19% vs 10%	<0.01
Carey [56]	2000	1953	uniquement si vaginose bactérienne	Vaginose bactérienne	métronidazole 2 g	16-24	12.2% vs 12.5%	ns
McDonald [57]	1997		uniquement si vaginose bactérienne	Vaginose bactérienne	métronidazole	24	7.2% vs 7.5%	ns
Hauth [58]	1995	624	antécédents d'accouchement prématuré	Vaginose bactérienne	erythromycine 5j métronidazole	22-24	26% vs 36%	0.01

[a] : Comparaison groupe « prise en charge » versus « pas de prise en charge ». La prise en charge diminue le risque d'accouchement prématuré que le prélèvement vaginal soit pathologique ou non

Tableau VII. Séquences nucléotidiques des amorces et sondes utilisées.

Mycoplasme étudié et amorce utilisée	Cible ou séquence nucléotidique	Taille (pb)
Ureaplasma spp.	Gène de l'urease	429
U4	ACGACGTCCATAAGCAACT	
U5	CAATCTGCTCGTGAAGTATTAC-biotin	
U9	GAGATAATGATTATATGTCAGGATCA	
M. hominis	16S rRNA	334
RNAH1	CAATGGCTAATGCCGGATACGC	
RNAH2	GGTACCGTCAGTCTGCAAT	
Contrôle de la spécificité		
U. urealyticum	Antigène gène MB	403 ou 448
UMS125	GTATTTGCAATCTTTATATGTTTTCG	
UMA226	CAGCTGATGTAAGTGCAGCATTAAATTC	

A partir de Stellrecht et coll [24].

Tableau VIII. CMI (mg/L) des antibiotiques vis-à-vis de, *M. hominis* et *Ureaplasma* spp.

Macrolides ou lincosamides	*M Hominis*	*Ureaplasma* spp.
Erythromycine	32 - >1000	0,02 - 4
Roxithromycine	>16 - > 64	0,06 - 4
Clarithromycine	16 - > 256	< 0,004 - 2
Azithromycine	4 - > 64	0,06 - 0,5
Josamycine	0,05 - 2	0,03 - 4
Spiramycine	32 - > 64	4 - 32
Midécamycine	0,25	ND
Clindamycine	< 0,008 - 2	0,2 - 64
Lincomycine	0,2 - 4	8 - 256

A partir de [34]

Figure 1: Localisations possibles de l'invasion bactérienne pendant la grossesse (d'après Goldenberg [3])

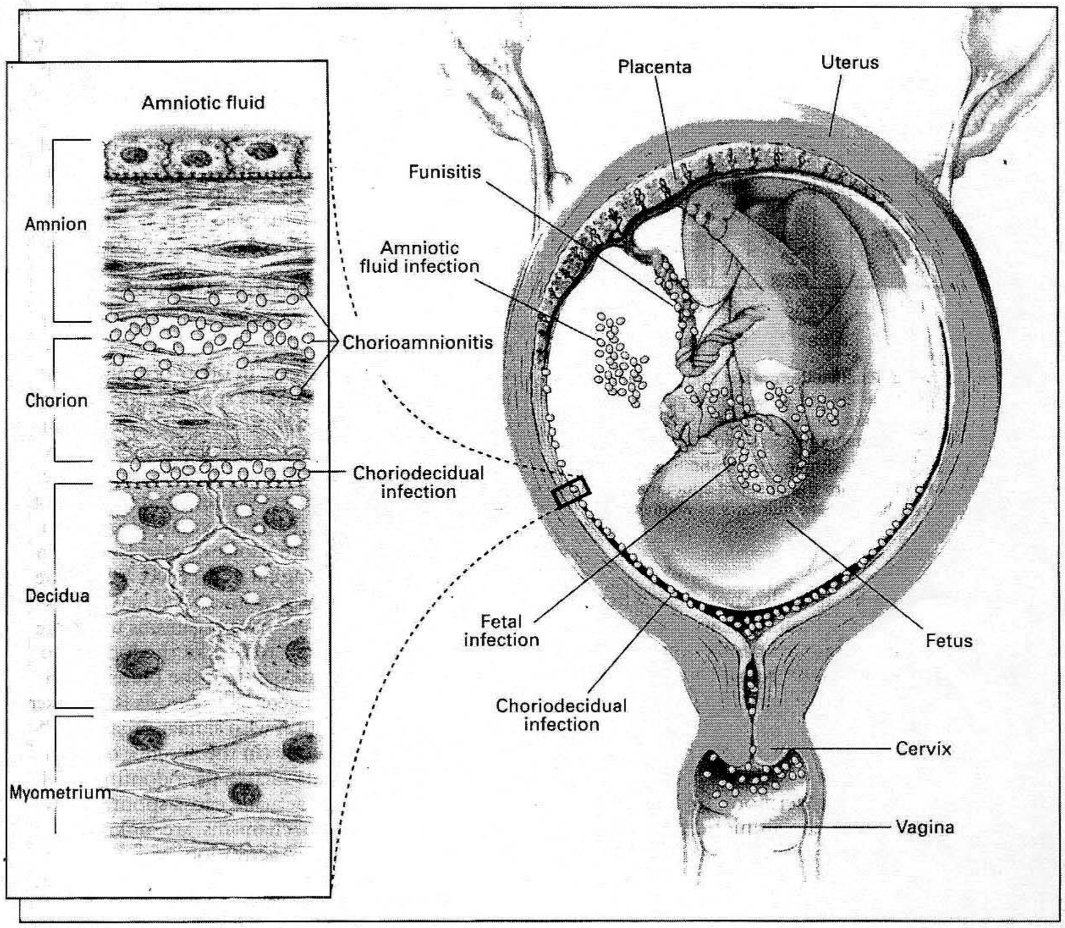

Références

1. BLONDEL B SK, MAZAUBRUN C, BREART G. Enquète nationale périnatale 2003 (INSERM: Unité de Recherche Epidémiologique en Santé périnatale et en Santé des femmes). Site internet: Direction Générale de la Santé, http://www.sante.gouv.fr/htm/dossiers/perinat03/sommaire.htm 2003.
2. LARROQUE B. [Developmental problems of very premature children at school age. Review of the literature]. J Gynecol Obstet Biol Reprod (Paris) 2004;33:475-86.
3. GOLDENBERG RL, HAUTH JC, ANDREWS WW. Intrauterine infection and preterm delivery. N Engl J Med 2000;342:1500-7.
4. WU YW. Systematic review of chorioamnionitis and cerebral palsy. Ment Retard Dev Disabil Res Rev 2002;8:25-9.
5. YOON BH, ROMERO R, MOON J, et al. Differences in the fetal interleukin-6 response to microbial invasion of the amniotic cavity between term and preterm gestation. J Matern Fetal Neonatal Med 2003;13:32-8.
6. YOON BH, ROMERO R, PARK JS, et al. Fetal exposure to an intra-amniotic inflammation and the development of cerebral palsy at the age of three years. Am J Obstet Gynecol 2000;182:675-81.
7. YOON BH, ROMERO R, KIM KS, et al. A systemic fetal inflammatory response and the development of bronchopulmonary dysplasia. Am J Obstet Gynecol 1999;181:773-9.
8. GOMEZ R, ROMERO R, GHEZZI F, YOON BH, MAZOR M, BERRY SM. The fetal inflammatory response syndrome. Am J Obstet Gynecol 1998;179:194-202.
9. DAMMANN O, KUBAN KC, LEVITON A. Perinatal infection, fetal inflammatory response, white matter damage, and cognitive limitations in children born preterm. Ment Retard Dev Disabil Res Rev 2002;8:46-50.
10. GONCALVES LF, CHAIWORAPONGSA T, ROMERO R. Intrauterine infection and prematurity. Ment Retard Dev Disabil Res Rev 2002;8:3-13.
11. KLEBANOFF MA, HILLIER SL, NUGENT RP, et al. Is bacterial vaginosis a stronger risk factor for preterm birth when it is diagnosed earlier in gestation? Am J Obstet Gynecol 2005;192:470-7.
12. HILLIER SL, NUGENT RP, ESCHENBACH DA, et al. Association between bacterial vaginosis and preterm delivery of a low-birth-weight infant. The Vaginal Infections and Prematurity Study Group. N Engl J Med 1995;333:1737-42.
13. LEITICH H, BODNER-ADLER B, BRUNBAUER M, KAIDER A, EGARTER C, HUSSLEIN P. Bacterial vaginosis as a risk factor for preterm delivery: a meta-analysis. Am J Obstet Gynecol 2003;189:139-47.
14. PARK KH, CHAIWORAPONGSA T, KIM YM, et al. Matrix metalloproteinase 3 in parturition, premature rupture of the membranes, and microbial invasion of the amniotic cavity. J Perinat Med 2003;31:12-22.
15. ROMERO R, CHAIWORAPONGSA T, ESPINOZA J, et al. Fetal plasma MMP-9 concentrations are elevated in preterm premature rupture of the membranes. Am J Obstet Gynecol 2002;187:1125-30.
16. MAYMON E, ROMERO R, PACORA P, et al. Evidence for the participation of interstitial collagenase (matrix metalloproteinase 1) in preterm premature rupture of membranes. Am J Obstet Gynecol 2000;183:914-20.
17. MAYMON E, ROMERO R, PACORA P, et al. Human neutrophil collagenase (matrix metalloproteinase 8) in parturition, premature rupture of the membranes, and intrauterine infection. Am J Obstet Gynecol 2000;183:94-9.

18. VADILLO-ORTEGA F, ESTRADA-GUTIERREZ G. Role of matrix metalloproteinases in preterm labour. Bjog 2005;112 Suppl 1:19-22.
19. WAITES KB, KATZ B, SCHELONKA RL. Mycoplasmas and ureaplasmas as neonatal pathogens. Clin Microbiol Rev 2005;18:757-89.
20. KUNDSIN RB, LEVITON A, ALLRED EN, POULIN SA. Ureaplasma urealyticum infection of the placenta in pregnancies that ended prematurely. Obstet Gynecol 1996;87:122-7.
21. CASSELL GH, WAITES KB, GIBBS RS, DAVIS JK. Role of Ureaplasma urealyticum in amnionitis. Pediatr Infect Dis 1986;5:S247-52.
22. YOON BH, ROMERO R, KIM M, et al. Clinical implications of detection of Ureaplasma urealyticum in the amniotic cavity with the polymerase chain reaction. Am J Obstet Gynecol 2000;183:1130-7.
23. GOLDSTEIN I, ZIMMER EZ, MERZBACH D, PERETZ BA, PALDI E. Intraamniotic infection in the very early phase of the second trimester. Am J Obstet Gynecol 1990;163:1261-3.
24. STELLRECHT KA, WORON AM, MISHRIK NG, VENEZIA RA. Comparison of multiplex PCR assay with culture for detection of genital mycoplasmas. J Clin Microbiol 2004;42:1528-33.
25. ABELE-HORN M, WOLFF C, DRESSEL P, et al. Polymerase chain reaction versus culture for detection of Ureaplasma urealyticum and Mycoplasma hominis in the urogenital tract of adults and the respiratory tract of newborns. Eur J Clin Microbiol Infect Dis 1996;15:595-8.
26. PERNI SC, VARDHANA S, KORNEEVA I, et al. Mycoplasma hominis and Ureaplasma urealyticum in midtrimester amniotic fluid: association with amniotic fluid cytokine levels and pregnancy outcome. Am J Obstet Gynecol 2004;191:1382-6.
27. GERBER S, VIAL Y, HOHLFELD P, WITKIN SS. Detection of Ureaplasma urealyticum in second-trimester amniotic fluid by polymerase chain reaction correlates with subsequent preterm labor and delivery. J Infect Dis 2003;187:518-21.
28. NGUYEN DP, GERBER S, HOHLFELD P, SANDRINE G, WITKIN SS. Mycoplasma hominis in mid-trimester amniotic fluid: relation to pregnancy outcome. J Perinat Med 2004;32:323-6.
29. KENYON S, BOULVAIN M, NEILSON J. Antibiotics for preterm rupture of the membranes: a systematic review. Obstet Gynecol 2004;104:1051-7.
30. KENYON SL, TAYLOR DJ, TARNOW-MORDI W. Broad-spectrum antibiotics for spontaneous preterm labour: the ORACLE II randomised trial. ORACLE Collaborative Group. Lancet 2001;357:989-94.
31. UGWUMADU A, MANYONDA I, REID F, HAY P. Effect of early oral clindamycin on late miscarriage and preterm delivery in asymptomatic women with abnormal vaginal flora and bacterial vaginosis: a randomised controlled trial. Lancet 2003;361:983-8.
32. SEEDS JW. Diagnostic mid trimester amniocentesis: how safe? Am J Obstet Gynecol 2004;191:607-15.
33. BEBEAR C, DE BARBEYRAC B, DEWILDE A, et al. [Multicenter study of the in vitro sensitivity of genital mycoplasmas to antibiotics]. Pathol Biol (Paris) 1993;41:289-93.
34. BEBEAR C. *Mycoplasma* et *Ureaplasma. In* Courvalin P, Leclercq R, Bingen C (ed.) Antibiogramme. EFKA Paris 2006 (à paraître).
35. WU YW, ESCOBAR GJ, GRETHER JK, CROEN LA, GREENE JD, NEWMAN TB. Chorioamnionitis and cerebral palsy in term and near-term infants. Jama 2003;290:2677-84.
36. PAPILE LA, BURSTEIN J, BURSTEIN R, KOFFLER H. Incidence and evolution of subependymal and intraventricular hemorrhage: a study of infants with birth weights less than 1,500 gm. J Pediatr 1978;92:529-34.
37. BELL MJ, TERNBERG JL, FEIGIN RD, et al. Neonatal necrotizing enterocolitis. Therapeutic decisions based upon clinical staging. Ann Surg 1978;187:1-7.

38. GRAVETT MG, HUMMEL D, ESCHENBACH DA, HOLMES KK. Preterm labor associated with subclinical amniotic fluid infection and with bacterial vaginosis. Obstet Gynecol 1986;67:229-37.
39. ROMERO R, EMAMIAN M, QUINTERO R, et al. The value and limitations of the Gram stain examination in the diagnosis of intraamniotic infection. Am J Obstet Gynecol 1988;159:114-9.
40. ROMERO R, SIRTORI M, OYARZUN E, et al. Infection and labor. V. Prevalence, microbiology, and clinical significance of intraamniotic infection in women with preterm labor and intact membranes. Am J Obstet Gynecol 1989;161:817-24.
41. ROMERO R, AVILA C, SANTHANAM U, SEHGAL PB. Amniotic fluid interleukin 6 in preterm labor. Association with infection. J Clin Invest 1990;85:1392-400.
42. ROMERO R, JIMENEZ C, LOHDA AK, et al. Amniotic fluid glucose concentration: a rapid and simple method for the detection of intraamniotic infection in preterm labor. Am J Obstet Gynecol 1990;163:968-74.
43. GAUTHIER DW, MEYER WJ, BIENIARZ A. Correlation of amniotic fluid glucose concentration and intraamniotic infection in patients with preterm labor or premature rupture of membranes. Am J Obstet Gynecol 1991;165:1105-10.
44. ROMERO R, QUINTERO R, NORES J, et al. Amniotic fluid white blood cell count: a rapid and simple test to diagnose microbial invasion of the amniotic cavity and predict preterm delivery. Am J Obstet Gynecol 1991;165:821-30.
45. COULTRIP LL, GROSSMAN JH. Evaluation of rapid diagnostic tests in the detection of microbial invasion of the amniotic cavity. Am J Obstet Gynecol 1992;167:1231-42.
46. WATTS DH, KROHN MA, HILLIER SL, ESCHENBACH DA. The association of occult amniotic fluid infection with gestational age and neonatal outcome among women in preterm labor. Obstet Gynecol 1992;79:351-7.
47. ROMERO R, YOON BH, MAZOR M, et al. The diagnostic and prognostic value of amniotic fluid white blood cell count, glucose, interleukin-6, and gram stain in patients with preterm labor and intact membranes. Am J Obstet Gynecol 1993;169:805-16.
48. COULTRIP LL, LIEN JM, GOMEZ R, KAPERNICK P, KHOURY A, GROSSMAN JH. The value of amniotic fluid interleukin-6 determination in patients with preterm labor and intact membranes in the detection of microbial invasion of the amniotic cavity. Am J Obstet Gynecol 1994;171:901-11.
49. YOON BH, YANG SH, JUN JK, PARK KH, KIM CJ, ROMERO R. Maternal blood C-reactive protein, white blood cell count, and temperature in preterm labor: a comparison with amniotic fluid white blood cell count. Obstet Gynecol 1996;87:231-7.
50. MARKENSON GR, MARTIN RK, TILLOTSON-CRISS M, FOLEY KS, STEWART RS, JR., YANCEY M. The use of the polymerase chain reaction to detect bacteria in amniotic fluid in pregnancies complicated by preterm labor. Am J Obstet Gynecol 1997;177:1471-7.
51. OYARZUN E, YAMAMOTO M, KATO S, GOMEZ R, LIZAMA L, MOENNE A. Specific detection of 16 micro-organisms in amniotic fluid by polymerase chain reaction and its correlation with preterm delivery occurrence. Am J Obstet Gynecol 1998;179:1115-9.
52. ROMERO R, QUINTERO R, OYARZUN E, et al. Intraamniotic infection and the onset of labor in preterm premature rupture of the membranes. Am J Obstet Gynecol 1988;159:661-6.
53. ROMERO R, YOON BH, MAZOR M, et al. A comparative study of the diagnostic performance of amniotic fluid glucose, white blood cell count, interleukin-6, and gram stain in the detection of microbial invasion in patients with preterm premature rupture of membranes. Am J Obstet Gynecol 1993;169:839-51.

54. AVERBUCH B, MAZOR M, SHOHAM-VARDI I, et al. Intra-uterine infection in women with preterm premature rupture of membranes: maternal and neonatal characteristics. Eur J Obstet Gynecol Reprod Biol 1995;62:25-9.
55. KISS H, PETRICEVIC L, HUSSLEIN P. Prospective randomised controlled trial of an infection screening programme to reduce the rate of preterm delivery. Bmj 2004;329:371.
56. CAREY JC, KLEBANOFF MA, HAUTH JC, et al. Metronidazole to prevent preterm delivery in pregnant women with asymptomatic bacterial vaginosis. National Institute of Child Health and Human Development Network of Maternal-Fetal Medicine Units. N Engl J Med 2000;342:534-40.
57. MCDONALD H, BROCKLEHURST P, PARSONS J. Antibiotics for treating bacterial vaginosis in pregnancy. Cochrane Database Syst Rev 2005:CD000262.
58. HAUTH JC, GOLDENBERG RL, ANDREWS WW, DUBARD MB, COPPER RL. Reduced incidence of preterm delivery with metronidazole and erythromycin in women with bacterial vaginosis. N Engl J Med 1995;333:1732-6.

Annexe I : Réalisation pratique de l'étude

Inclusion : Amniocentèse et prélèvement de 3 ml de liquide amniotique entre 15 et 20 SA.
Explication de l'étude et du suivi associé
Consentement signé par la patiente

Saisie sur un CRF électronique informatisé accessible à tous les centres

Envoi des prélèvements au jour le jour avec une conservation à 4°C

Délai : 24 h

Service de bactériologie de l'hopital Cochin
Télécopie du résultat à l'URC d'Henri Mondor

Délai : 24-72 h

Randomisation et attribution d'un numéro de lot de traitement directement sur cleanweb si PCR + pour *Mycoplasma hominis* ou *Ureaplasma* spp.
Le technicien de recherche clinique téléphone à la patiente et lui propose un rendez vous avec le médecin.
Randomisation en consultation sur le logiciel cleanweb.
Communication du numéro de lot de traitement à la pharmacie du centre investigateur et à l'investigateur

Délai : 1 à 7 jours

Ordonnance de traitement remise à la patiente

Délai total prévu avant la réception du traitement par la patiente : 5-12 jours

Annexe II : Contrôle téléphonique de l'observance au traitement et de la survenue d'effets secondaires

Contrôle effectué à J15 après l'administration du traitement.

1- Avez-vous pris le traitement pendant 10 jours à la dose prescrite ?

2- Avez-vous ressenti un symptôme inhabituel pendant cette période ?

3- Avez-vous eu des nausées, des douleurs, une diarrhée ou des boutons ?

4- Combien de comprimés reste-t-il dans les plaquettes qui vous ont été fournies ?

Annexe III : Fiche de déclaration des EIGs :

FORMULAIRE DE DECLARATION D'UN EVENEMENT NDESIRABLE GRAVE (EIG) SUSCEPTIBLE D'ETRE DU A UNE RECHERCHE BIOMEDICALE SUR UN MEDICAMENT OU PRODUIT ASSIMILE

ASSISTANCE PUBLIQUE HÔPITAUX DE PARIS

DEPARTEMENT de la RECHERCHE CLINIQUE et du DEVELOPPEMENT DIRC Ile-de-FRANCE

PARTIE RESERVEE AU PROMOTEUR : NE PAS REMPLIR
|__|__| - |__|__| - *DRCD* - |__|__|__|__|__| - |__|__|__|__|

Cette fiche doit être retournée dûment complétée (2 pages) au DRCD par fax : +33 (0)1 44 84 17 99
A l'attention de Christophe AUCAN et de Shohreh AZIMI

Date de notification : |__|__| jj |__|__| mm |__|__|__|__| aaaa

Code de la Recherche : *P060216 - PREMYC*

N° EudraCT : 2006-005336-24 **Déclaration initiale** ☐ **Suivi d'EIG déclaré** ☐

Titre de la Recherche Biomédicale : *Réduction de la prématurité spontanée : Impact d'un traitement antibiotique en cas de PCR positive pour Ureaplasma spp. et/ou Mycoplasma hominis dans le liquide amniotique*

1) Nom et adresse du centre : ______________________________

Centre n° : |__|__| Investigateur (Qualité - Nom - Prénom) : ______________________________

2) Identification du patient :

Nom : |__|__|__| Prénom : |__|__|

Patient n° : |__|__|__|__|__|

Sexe : Masculin ☐ Féminin ☐

Date de naissance : |__|__| |__|__| |__|__|__|__|

Age : |__|__| ans

Poids : |__|__|__| kg

Taille : |__|__|__| cm

Date d'inclusion : |__|__| |__|__| |__|__|__|__|

Date de randomisation : |__|__| |__|__| |__|__|__|__|

Bras ____________ ☐ Bras ____________ ☐

3) Evénement indésirable grave :

Décès ☐

Mise en jeu du pronostic vital ☐

Nécessite ou prolonge l'hospitalisation : ☐
Du |__|__||__|__||__|__| *au* |__|__||__|__||__|__| ☐ *en cours*

Incapacité ou invalidité ☐

Anomalie congénitale ☐

Autre(s) critère(s) médicalement significatif(s) (préciser) : ☐

____

____

Antécédents (allergie, insuffisance rénale …) : ______________________________

____

4) Description complète de l'événement indésirable (diagnostic retenu, localisation anatomique, critères permettant de considérer l'événement comme grave) :

Intensité : Légère ☐ Modérée ☐ Sévère ☐

Date de survenue : |__|__| jj |__|__| mm |__|__|__|__| aaaa et heure de survenue : |__|__| hh |__|__| min

____

____

____

Délai de survenue après la dernière prise : ______________________________

5) Médicament(s) expérimental(aux) administré(s) avant la survenue de l'événement indésirable :

Nom commercial (de préférence) ou Dénomination Commune Internationale	Voie	Dose/ 24h	Date de début	En cours	Date de fin	Indication	Causalité * (1,2,3 ou 4)
			|__|__| |__|__| |__|__|	☐	|__|__| |__|__| |__|__|		
			|__|__| |__|__| |__|__|	☐	|__|__| |__|__| |__|__|		
			|__|__| |__|__| |__|__|	☐	|__|__| |__|__| |__|__|		
			|__|__| |__|__| |__|__|	☐	|__|__| |__|__| |__|__|		

** 1 = Probable 2 = Possible 3 = Non liée 4 = Inconnue*

6) Médicament(s) concomitant(s) à l'exclusion de ceux utilisés pour traiter l'événement indésirable :

Nom commercial (de préférence) ou Dénomination Commune Internationale	Voie	Dose/ 24h	Date de début	En cours	Date de fin	Indication	Causalité * (1,2,3 ou 4)
			|__|__| |__|__| |__|__|	☐	|__|__| |__|__| |__|__|		
			|__|__| |__|__| |__|__|	☐	|__|__| |__|__| |__|__|		
			|__|__| |__|__| |__|__|	☐	|__|__| |__|__| |__|__|		
			|__|__| |__|__| |__|__|	☐	|__|__| |__|__| |__|__|		

** 1 = Probable 2 = Possible 3 = Non liée 4 = Inconnue*

7) Evolution (indiquez si des mesures symptomatiques ont été prises : non ☐ oui ☐ *Si oui, préciser*) :

____

____

8) Date de disparition : |__|__| |__|__| |__|__|__|__| et heure de disparition : |__|__| |__|__|

jj mm aaaa hh min

9) Autre(s) étiologie(s) envisagée(s) : non ☐ oui ☐ *Si oui, préciser :*

____

____

10) Examen(s) complémentaire(s) réalisé(s) : non ☐ oui ☐ *Si oui, préciser date, nature et résultats :*

____

____

11) Traitements de la Recherche Biomédicale :

Levée d'insu : non ☐ oui ☐ non applicable ☐ date : |__|__| |__|__| |__|__|__|__|

Résultat de la levée d'insu : ____

Ré-administration du (des) médicament(s) : non ☐ oui ☐ non applicable ☐ date : |__|__| |__|__| |__|__|__|__|

Si oui, le(s)quel(s) : ____

Récidive après ré-administration : non ☐ oui ☐ non applicable ☐ date : |__|__| |__|__| |__|__|__|__|

12) Selon l'investigateur, l'événement indésirable grave semble plutôt lié :

☐ au(x) médicament(s) de la recherche : le(s)quel(s) : ______________________ ☐ à une maladie intercurrente

☐ au(x) médicament(s) concomitant(s) : le(s)quel(s) : ______________________ ☐ à la progression de la maladie

☐ aux procédures de la recherche biomédicale ☐ autre : ______________

Date : |__|__| |__|__| |__|__| **Tampon du service :** **Nom :** ____________ **Signature :**

PARTIE RESERVEE AU PROMOTEUR : NE PAS REMPLIR

***Numéro d'identification de l'événement :* EV I__I__I__I**

Date de réception par le promoteur : |__|__| |__|__| |__|__|__|__|

Date de ce rapport : |__|__| |__|__| |__|__|__|__| ☐ initial ☐ suivi n° |__|__|

Selon le promoteur, l'événement indésirable semble plutôt lié :

☐ au(x) médicament(s) de la recherche : le(s)quel(s) : ______________________ ☐ à une maladie intercurrente

☐ au(x) médicament(s) concomitant(s) : le(s)quel(s) : ______________________ ☐ à la progression de la maladie

☐ aux procédures de la recherche biomédicale ☐ autre : ______________

Si selon le promoteur, l'événement semble plutôt lié au médicament :

☐ L'événement indésirable grave est attendu ☐ L'événement indésirable grave est inattendu

Commentaires du promoteur : ____

____

____

Nom et qualité du représentant du promoteur : **Signature**
