# Supplementary material for: Antibiotics for amniotic-fluid colonization by Ureaplasma and/or Mycoplasma spp. to prevent preterm birth: A randomized trial
Source: PLoS One. 2018 Nov 7;13(11):e0206290. doi: 10.1371/journal.pone.0206290 (PMC6221323; doi:10.1371/journal.pone.0206290)
Supplement: S3 File — (PDF) [file pone.0206290.s005.pdf]

Kit Ref. No.: DICD-XX-L100*

Version: 02
Last revision date: 7/07/2016

# DiaControlDNA™

## DNA extraction and real-time PCR inhibition control

## Instructions for Use

*Available dyes and corresponding reference numbers:

- Yellow Dye: DICD-YD-L100
- Dragon Dye: DICD-DR-L100
- Texas Red: DICD-TR-L100
- Cy5: DICD-CY-L100
- Red Dye: DICD-RD-L100

CE

diagenode

## Revision notes compared to the previous version:

**Previous version: Version 01 (7/01/2015)**

| Revision date | Description |
|---|---|
| 2/08/2016 | • Changed the kit's name and reference number:<br>- Old reference number: DIA-EIC/DNA-050<br>- New reference number: DICD-XX-L100<br>• New color for reagent tube caps (see section 3.5)<br>• New Instructions for Use layout<br>• New reference code for downloading this user manual from the website www.e-labeling.eu/dgn: DICD-XX-V02 |
| 7/07/2016 | • Removed the SGS logo |

# Contents

1. GLOSSARY ........ 1

2. GENERAL INFORMATION ........ 2

2.1 Intended Use ........ 2
2.2 Pathogen Information ........ 2
2.3 Product Description ........ 2
2.4 Target Sequences ........ 3
2.5 Kit Contents ........ 3

3. REAGENT STORAGE, HANDLING, AND STABILITY ........ 4

4. REQUIRED MATERIALS (NOT SUPPLIED) ........ 5

5. WARNINGS AND PRECAUTIONS ........ 6

6. SPECIMEN COLLECTION, STORAGE, AND TRANSPORT ........ 7

7. PROTOCOL ........ 8

Extraction ........ 8
Master mix preparation ........ 9
Real-time PCR system setup ........ 10

7. INTERPRETATION OF RESULTS ........ 11

8. TROUBLESHOOTING GUIDE ........ 12

Nucleic acid isolation ........ 12
PCR (weak or no signal from the extracted DNA and/or EIC) ........ 12
PCR system failure ........ 12

9. PERFORMANCE EVALUATION ........ 13

9.1 Analytical Sensitivity ........ 13
9.2 Analytical Specificity ........ 13

10. LIMITATIONS ........ 14

11. QUALITY CERTIFICATION ........ 14

12. REFERENCES ........ 14

13. EXPLANATION OF SYMBOLS ........ 15

14. NOTICE TO PURCHASER ........ 16

# 1. Glossary

| | |
|---|---|
| BAL | Bronchoalveolar lavage |
| $C^-$ | Negative control |
| $C^+$ | Positive control |
| Cp | Crossing point |
| Ct | Cycle threshold |
| DNA | Deoxyribonucleic acid |
| DR | Dragonfly orange dye |
| EIC | Extraction and inhibition control |
| Em. | Emission |
| Ex. | Excitation |
| GLP | Good Laboratory Practice |
| IVD | *In vitro* diagnostics |
| LC | LightCycler |
| LoD | Limit of Detection |
| MM | Master Mix |
| PBS | Phosphate Buffered Saline |
| PCR | Polymerase chain reaction |
| PP | Probe and Primers |
| $PP_{EIC}$ | EIC probe and primers |
| PPp | Pathogen probe and primers |
| qPCR | Quantitative polymerase chain reaction |
| RD | Red dye |
| RNA | Ribonucleic acid |
| STM | Specimen Transport Medium |
| TR | Texas Red dye |
| YD | Yellow dye |

# 2. General Information

## 2.1 Intended Use

The DNA extraction and real-time PCR inhibition control is an *in vitro* diagnostic method. This method must be carried out by qualified technicians in diagnostic laboratories.

The DNA extraction & real-time PCR inhibition control was validated for use with:

- ABI ® (7000 / 7500)
- Roche LightCycler® 480
- Biorad® iCycler/ CFX96/ Dx

The kit is compatible with most real-time PCR systems (details on request):

- ABI ® 7300 / 7900HT/ StepOnePlus™
- Roche LightCycler® 2.0
- Biorad® IQ5
- Qiagen Rotor-Gene® 3000 / 6000
- Cepheid SmartCycler® II

## 2.2 Pathogen Information

The DNA extraction & inhibition control is a DNA virus exhibiting no sequence similarity to the human genome, viruses, or other pathogens affecting humans.

The DNA extraction & inhibition control is a complete virus that has retained its ability to cause infection. This virus is classified by the EFB (European Federation of Biotechnologies) as a Ea1* microorganism.

It is not considered to be dangerous to humans, but must be handled under a laminar flow hood. (* More information is provided at this end of this manual).

The Diagenode DNA extraction & inhibition control is a viral culture with a viral load of 1,000 TCID50/mL.

## 2.3 Product Description

This product consists of a DNA Extraction and real-time PCR Inhibition control. The DNA Extraction and Inhibition Control has been validated primarily for use with respiratory, stool, blood, and urine samples.

Various fluorescent dyes are available for this PCR control:

- Yellow Dye (emission 530/549 nm), ref. no.: DICD-YD-L100
- Orange Dye (emission 554/576 nm), ref. no.: DICD-DR-L100
- Texas Red Dye (emission 583/603 nm), ref. no.: DICD-TR-L100
- Cy5 Dye (emission 646/662 nm), ref. no.: DICD-CY-L100
- Red Dye (emission 644/669 nm), ref. no.: DICD-RD-L100

## 2.4 Target Sequences

❖ The **primers** were specially designed to hybridize to a specific sequence in the target pathogen.

| Target pathogen | Target gene |
|---|---|
| Phocid Herpesvirus | Glycoprotein B |

❖ The **probe** was designed to hybridize to a specific sequence in the target pathogen. It includes a fluorescent reporter dye at the 5' end, whose fluorescence is quenched by a second dye at the 3' end. The probe only emits a signal if it is bound to the amplified product.

Several fluorescent dyes are available for detection of the target gene:

| Control References | Reporter Dye |
|---|---|
| DICD-YD-L100 | YELLOW (Ex./Em. 530/549 nm) |
| DICD-DR-L100 | DRAGONFLY ORANGE (Ex./Em 554/576 nm) |
| DICD-TR-L100 | TEXAS RED (Ex./Em. : 583/603 nm) |
| DICD-CY-L100 | CY5 (Ex./Em.662/646 nm) |
| DICD-RD-L100 | RED DYE (Ex./Em. 644/669 nm) |

## 2.5 Kit Contents

This kit contains the following reagents:

| Reagents pouch | | | |
|---|---|---|---|
| Reagents | Cap color | Volume | Quantity |
| Primers and hydrolysis probe | Red | 250 µL | 1 tube |
| Viral culture (DNA) | Yellow | 1000 µL | 1 tube |

The kit contains enough reagents to perform 100 reactions with a final volume of 25 µL.

The Master Mix is not included in the kit.
Recommended Master Mixes:

- 2x: LC 480 Probe Master, Roche (ref. no.: 4707494001)
- 5x: Optima Du Master Mix 5X DNA, Diagenode (DMML-D5-D100)
- TaqMan Probe LC 2.0 for LightCycler 2.0, Roche (ref. no.: 4535286001)

Other validated Master Mixes:

- 2x: QuantiFast Multiplex + R kit (2000), Qiagen (ref. no.: 204756)
- QuantiFastTM Probe PCR + ROX Vial Kit, Qiagen (ref. no.: 204354)
- Rotor-Gene Multiplex PCR kit for Qiagen Rotor-Gene (ref. no.: 204774)

# 3. Reagent Storage, Handling, and Stability

| Condition | Storage temperature | Stability |
|---|---|---|
| Unopened reagent | ≤ -20°C | Expiration date printed on the label |

- ✓ Store reagents in the dark and protect from light.
- ✓ Keep all kit contents on ice during use.
- ✓ Storing the kit at room temperature can cause its contents to degrade, thereby reducing sensitivity. If this should occur, use a new kit.
- ✓ Kits are shipped frozen and should arrive frozen. Store at T ≤ -20°C after receipt.
- ✓ Avoid repeated freeze-thaw cycles. Prepare aliquots if necessary.

# 4. Required Materials (not supplied)

## Disposables

- Sterile pipette tips with filter (DNase-/RNase-free)
- 1.5 mL and/or 2 mL microcentrifuge tubes (DNase-/RNase-free)
- 96-well reaction plate and adhesive film / PCR tubes / capillaries

## Laboratory Equipment

- Biosafety cabinet, laminar flow hood
- Cooling block/ice
- Pipettes (accuracy range between 1-1000 µL)
- Vortex mixer
- Microcentrifuge
- Real-time PCR system: ABI / Roche / Bio- Rad / Qiagen / Agilent Stratagene / Cepheid

## Reagents

- DNA isolation kit (depending on the extraction system used)
- $H_2O$ (PCR grade)
- Recommended Master Mixes:
  - 2x: LC 480 Probe Master, Roche (ref. no.: 4707494001)
  - 5x: Optima Du Master Mix 5X DNA, Diagenode (DMML-D5-D100)
  - TaqMan Probe LC 2.0 for LightCycler 2.0, Roche (ref. no.: 4535286001)
- Other validated Master Mixes:
  - 2x: QuantiFast Multiplex + R kit (2000), Qiagen (ref. no.: 204756)
  - QuantiFastTM Probe PCR + ROX Vial Kit, Qiagen (ref. no.: 204354)
  - Rotor-Gene Multiplex PCR kit for Qiagen Rotor-Gene (ref. no.: 204774)

# 5. Warnings and Precautions

**If the package is damaged or if the kit has thawed,**
**please contact the logistics department at:**

☎ **logistics@diagenode.com / +32 4 364 20 52**

- Read all instructions before using the kit.
- Do not use reagents if the protective case has been opened or damaged upon arrival.
- Do not use reagents if the tubes have been opened or damaged.
- Do not use reagents and/or materials beyond their expiration date.
- Do not substitute reagents from one kit with reagents with a different batch number or from other manufacturers.
- This product must be used by laboratory personnel with training in real-time PCR techniques.
- Always follow good laboratory practices, such as:
    - Maintain separate laboratory areas for nucleic acid extraction and for real-time amplification. The PCR laboratory (biosafety cabinet, DNA/RNA extraction platform, work surfaces, etc.) must be cleaned and disinfected with the appropriate cleaning solutions after each PCR experiment.
    - Conduct quality control as discussed in section 4.
    - Wear lab coats and disposable gloves when handling kit reagents. Wash hands thoroughly when finished performing the test.
    - Close all tubes and vials after use. Do not mix up the tube caps or reuse caps. This may cause contamination and compromise the test results.
    - Use only tips with filters for all PCR mixes.
- Always handle viruses (such as the one for the internal control) inside a biosafety cabinet when performing a manual extraction.
- Anyone showing symptoms of the diseases targeted by the kit should not use it.
- Dispose of the kit if contamination is suspected.
- After use, waste, materials, and reagents must be considered infectious and be disposed of in appropriately designated biological waste containers. Dispose of any unused reagents and waste according to local regulations.
- Always handle specimens as if infectious using safe laboratory procedures in accordance with applicable local regulations.
- Do not pipette by mouth. Do not ingest any of the kit contents.
- Do not smoke, drink, or eat in areas where specimens and/or kit reagents are being handled.

# 6. Specimen Collection, Storage, and Transport

Diagnostics by PCR is dependent upon the quality of the specimen collection, their timely delivery to the laboratory in proper containers, and storage under the appropriate conditions before analysis.

| Type | Collection | Storage/Transport* |
|---|---|---|
| Respiratory samples (BAL fluid, sputum, nasopharyngeal aspirates / swabs), Blood, Plasma, Urine, Stool | Collect and transport samples in DNase-/RNase-free polypropylene tubes** | Store human samples at 4°C for up to 24 hours, or at -20°C or -80°C for later use.<br>Transport human samples at -20°C. |

*Human samples must be transported according to the required regulations for the transport of potentially infectious substances.

**To ensure ideal storage and transport conditions, follow manufacturer instructions for containers and transport medium.

Repeated freezing and thawing of human samples can compromise PCR sensitivity.

# 7. Protocol

## Workflow

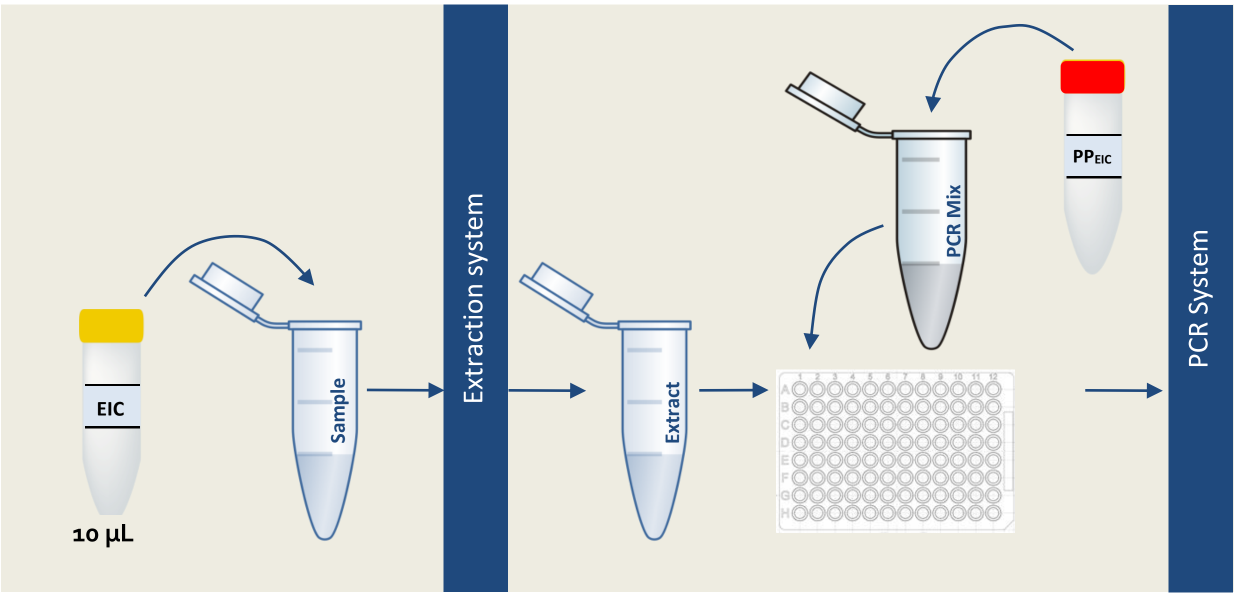


Note:
Prepare a new mix for each new PCR assay. The Master Mix (not supplied) and the primers/probes provided in the kit must be kept at 4°C throughout the entire PCR preparation stage.

## Extraction

1. Thaw the tube (yellow cap) containing the viral culture (EIC) on ice.
2. Add 10 µL of viral culture (EIC) to each sample prior to extraction.
3. Vortex the tube containing the sample and the viral culture (EIC).
4. Follow the instructions for extraction according to the extraction method used.

The extraction and inhibition control has been validated for use with respiratory, stool, blood, and urine samples.

Diagenode recommends several extraction methods to avoid PCR inhibitors as much as possible:

- NucliSENS easyMag®
- MagNa Pure LC® system
- QIASymphony®
- QIAamp® DNA Mini Kit (ref. no.: 51304)
- QIAamp® DNA Blood Mini Kit (ref. no.: 51104)
- QiAamp® DNA Micro Kit (56304)
- QiAamp® DNA stool Mini Kit (51504)
- Maxwell® 16 Blood DNA Purification Kit (ref. no.: AS1010)

## Master mix preparation

Recommended Master Mixes:

- 2x: LC 480 Probe Master, Roche (ref. no.: 4707494001)
- 5x: Optima Du Master Mix 5X DNA, Diagenode (DMML-D5-D100)
- TaqMan Probe LC 2.0 for LightCycler 2.0, Roche (ref. no.: 4535286001)

Other validated Master Mixes:

- 2x: QuantiFast Multiplex + R kit (2000), Qiagen (ref. no.: 204756)
- QuantiFastTM Probe PCR + ROX Vial Kit, Qiagen (ref. no.: 204354)
- Rotor-Gene Multiplex PCR kit for Qiagen Rotor-Gene (ref. no.: 204774)

Protocol examples:

| 25 µL PCR | |
|---|---|
| MM 2X | 12.5 µL |
| $PP_P$ | 2.5 µL |
| $PP_{EIC}$ | 2.5 µL |
| H2O (PCR grade) | 2.5 µL |
| Extracted sample / Positive control / Negative control | 5 µL |
| Final volume | **25 µL** |

| 25 µL PCR | |
|---|---|
| MM 5X | 5 µL |
| $PP_P$ | 2,5 µL |
| $PP_{EIC}$ | 2,5 µL |
| H2O (PCR grade) | 10 µL |
| Extracted sample / Positive control / Negative control | 5 µL |
| Final volume | **25 µL** |

| 50 µL PCR | |
|---|---|
| MM 2X | 25 µL |
| $PP_P$ | 5 µL |
| $PP_{EIC}$ | 5 µL |
| H2O (PCR grade) | 5 µL |
| Extracted sample / Positive control / Negative control | 10 µL |
| Final volume | **50 µL** |

| 50 µL PCR | |
|---|---|
| MM 5X | 10 µL |
| $PP_P$ | 5 µL |
| $PP_{EIC}$ | 5 µL |
| H2O (PCR grade) | 20 µL |
| Extracted sample / Positive control / Negative control | 10 µL |
| Final volume | **50 µL** |

| 20 µL PCR* | |
|---|---|
| MM 5X | 4 µL |
| $PP_P$ | 2 µL |
| $PP_{EIC}$ | 2 µL |
| H2O (PCR grade) | 7 µL |
| Extracted sample / Positive control / Negative control | 5 µL |
| Final volume | **20 µL** |

*Protocol for LC2.0 (Roche)

## Real-time PCR system setup

1. Select the detection channels for the PCR system and dyes being used.

| | FAM | YD | DR | TR | CY5 | RD |
|---|---|---|---|---|---|---|
| Excitation (nm) | 494 | 530 | 554 | 583 | 646 | 644 |
| Emission (nm) | 520 | 549 | 576 | 603 | 662 | 669 |
| **PCR System** | | | | | | |
| **ABI® *** | | | | | | |
| 7000 / 7300 / 7900HT | FAM | VIC | NED | ROX | | |
| 7500 | FAM | VIC | NED | TR | CY5 | CY5 |
| StepOne™ | FAM | VIC | | ROX | | |
| StepOnePlus™ | FAM | VIC | NED | ROX | | |
| **Bio-Rad** | | | | | | |
| iCycler /CFX96™ / IQ™5 | FAM | HEX | | TR | CY5 | CY5 |
| **Roche **** | | | | | | |
| LightCycler® 2.0 | 530 | 560 | | | | |
| LightCycler® 480 | 510 465 | 580 533 | | 610 533 | 660 618 | 660 618 |
| **Qiagen***** | | | | | | |
| Rotor-Gene® 3000 (Corbett) | FAM | JOE | | ROX | CY5 | CY5 |
| Rotor-Gene Q (Corbett Rotor-Gene 6000) | Green | Yellow | | Orange | Red | Red |
| **Stratagene ***** | | | | | | |
| Mx 3005P™ / Mx3000P® | FAM | HEX | | TR | CY5 | CY5 |
| **Cepheid** | | | | | | |
| SmartCycler® II | FAM | Alexa 532 | | TR | | Alexa 647 |

* For ABI systems:
- Select "None" for the quencher
- Select "Passive reference: ROX" if including ROX in the master mix
- Select "Passive reference: none" if not including ROX in the master mix

** Roche systems: You must create a color compensation object (.ccc file) for each master mix used. (Diagenode, ref. no.: DMAF-L4)

*** Stratagene systems: In "Filter Set Gain Settings", select under FAM: X8, for Yellow dye: X2, for CY5: X4.

2. Run the PCR following the temperature protocol shown below:

For ABI 7000-7300-7500-7900HT-StepOnePlus™ / Roche LightCycler® 480 / Bio-Rad iCycler-IQ5-CFX96 / Agilent Stratagene MX3000P-3005P / Qiagen Rotor-Gene® 3000- Qiagen Rotor-Gene® 6000 / Cepheid SmartCycler® II

| | PCR steps | | | |
|---|---|---|---|---|
| | Preparation | Activation (Taq) | Amplification | |
| Temperature | 50°C | 95°C | 95°C | 60°C |
| Time | 2 min. | 10 min. | 15 sec. | 60 sec. |
| Cycles | 1 | 1 | 45 | |
| Ramp rate | - | - | 50% | 50% |

For the Roche LC2.0 system

| | PCR steps | | | | |
|---|---|---|---|---|---|
| | Activation (Taq) | Amplification | | | Cool down |
| Temperature | 95°C | 95°C | 60°C | 72°C | 40°C |
| Time | 10 min. | 10 sec. | 40 sec. | 1 | 30 sec. |
| Cycles | 1 | 45 | | | 1 |
| Ramp rate | - | - | - | - | - |

Note:
To ensure the PCR system is properly set up, refer to the user manual for the target pathogen kit.

# 7. Interpretation of Results

| Detected signal:<br>Yellow Dye<br>Orange Dye<br>Texas Red<br>Cy5<br>Red Dye | Interpretation of Results |
|---|---|
| The signal is **above** the detection threshold | The sample is positive for the EIC DNA<br>The PCR assay is valid |
| The signal is **below** the detection threshold | The sample is negative for the EIC DNA<br>The PCR assay is not valid* |

*If the sample tested has a signal above the detection threshold, the PCR assay is valid even if the signal from the EIC is below the detection threshold.

# 8. Troubleshooting Guide

## Nucleic acid isolation

- Refer to the extraction system's user manual
- Perform preventative maintenance on automatic extraction systems (see user manual)
- Verify the reagent expiration dates and storage conditions

## PCR (weak or no signal from the extracted DNA and/or EIC)

- Verify the extraction procedure The procedure should be validated and minimize the presence of inhibitors
- Verify the PCR protocol For best results, use the volumes described in this manual (section 7 Protocol) as well as the recommended master mixes
- Verify the calibration of the pipettes (according to manufacturer's recommendations)
- Verify the reagent expiration dates and storage conditions
- Verify the software program and thermal profile for the PCR reaction
- Verify the dyes selected (depending on the PCR system)

## PCR system failure

- Refer to the PCR system's user manual

# 9. Performance Evaluation

## 9.1 Analytical Sensitivity

Performance of the DiaControlDNA™ kit was evaluated by testing 137 biological samples (BAL fluid, sputum, nasopharyngeal aspirates/swabs).

The DNA Extraction & Inhibition control was added to each sample before extraction, and the biological samples were extracted using the NucliSENS easyMAG (Biomerieux). Nucleic acid amplification was performed in the ABI 7000 system.

The following results were obtained:

| N (samples) = 93 | Ct |
|---|---|
| **Mean of the results** | 32.24 |
| **Standard deviation** | 1.54 |
| **Minimum** | 27 |
| **Maximum** | 35 |

The same results were obtained using the extraction procedure with the Qiagen DNA blood Miniprep Kit.

## 9.2 Analytical Specificity

The specificity of the DiaControlDNA™ kit was achieved primarily by selecting the primers and probe from literature as well as the very strict PCR reaction conditions. The primers and probe were verified by aligning the sequences to all the sequences published in the DNA databases to identify possible homologies.

In order to determine the assay specificity of the DiaControlDNA™ kit, the pathogens listed in the table below were tested for potential cross-reactivity.

| Bacteria | | |
|---|---|---|
| *Bartonella* | *Legionella pneumophila* | *Staphylococcus aureus* |
| *Bordetella* | *Listeria monocytogenes* | *Streptococcus B* |
| *Borrelia burgdorferi* | *Mycoplasma pneumoniae* | *Streptococcus pneumoniae* |
| *Chlamydophila pneumoniae* | *Neisseria gonorrhoeae* | *Treponema pallidum* |
| *Haemophilus influenzae* | *Neisseria meningitidis* | |
| **Viruses** | | |
| Adenovirus | HBV | Parvovirus |
| CMV | Herpes | Varicella zoster |
| **Parasites** | | |
| *Toxoplasma gondii* | | |
| **Fungi** | | |
| *Aspergillus* | *Candida(s)* | |

**None of the pathogens tested showed any cross reactivity.**

# 10. Limitations

- All reagents must only be used for *in vitro* diagnostic testing.
- Strict adherence to the instructions for use is necessary for optimum results.
- The reliability of results is dependent upon proper specimen collection, transport, storage, and processing procedures.
- This test has been validated for use with the ABI / Roche / Bio-Rad / Qiagen / Agilent / Stratagene / Cepheid PCR systems.
- The extraction and inhibition control has been validated for use with respiratory, stool, blood, and urine samples.
- False negatives may also occur if nucleic acids have degraded before using the test. The inhibition control provides the ability to identify samples containing PCR amplification inhibitors. However, this control does not indicate whether or not the nucleic acid has degraded resulting from improperly collected, transported, or stored specimens.
- False positives may occur due to cross-contamination between the target organism and nucleic acids or amplicons, or due to non-specific signals in the test.

# 11. Quality Certification

Diagenode has received ISO 9001 and ISO 13485 certification for the design, manufacture, and sale of *in vitro* diagnostic medical devices (IVDMD) for infectious diseases using nucleic acid technology.

The DiaControlDNA™ kit has been tested against predetermined specifications to ensure product quality.

# 12. References

(1) Templeton KE, Scheltinga SA, vander ZEE A., Diederen BM, van Krijssen AM, Goossens H, Kuiper E., Claas EC (2003) Evaluation of real-time PCR for detection of and discrimination between *Bordetella pertussis*, *Bordetella parapertussis* and *Bordetella holmesii* for clinical diagnosis. *Journal of Clinical Microbiology*, 41(9):4121-4126

(2) Eric C. J. Claas, Marco W. Schilham, Caroline S. de Brouwer, Petr Hubacek, Marcela Echavarria, Arjan C. Lankester, Maarten J. D. van Tol, et Aloys C. M. Kroes (2005) Internally Controlled Real-Time PCR Monitoring of Adenovirus DNA Load in Serum or Plasma of Transplant Recipients. *Journal of Clinical Microbiology*, 43(4):1738-1744.

# 13. Explanation of Symbols

| | |
|---|---|
| REF | Catalog reference number |
| LOT | Batch code |
| 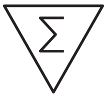 | Contains sufficient for "n" tests |
| 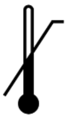 | Upper temperature limit |
| 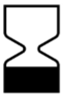 | Use by date (yyyy-mm) |
| IVD | *In vitro* diagnostic medical device |
| 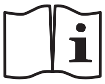 | Refer to user manual |
| 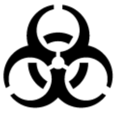 | Biological hazard |
| CONTROL | Control |
| CONTROL – | Negative Control |
| CONTROL + | Positive Control |
| 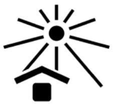 | Store in the dark |
| 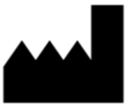 | Manufacturer |

**Europe Diagenode sa** / LIEGE SCIENCE PARK // Rue du Bois Saint-Jean, 3 // 4102 Seraing // Belgium //
Telephone: (+32) 4 364 20 50 // Email: info@diagenodediagnostics.com

# 14. Notice to Purchaser

This product has been optimized for use in the polymerase chain reaction (PCR) covered by patents owned by Roche Molecular Systems, Inc. and F. Hoffmann-La Roche ltd. (Roche). No license under these patents to use the PCR process is conveyed expressly or by implication to the purchaser by the purchase of this product.
A license to use the PCR process for certain research and development activities accompanies the purchase of certain reagents from licensed suppliers, when used in conjunction with an authorized thermal cycler, or is available from Applied Biosystems. Further information on purchasing licenses to practice the PCR process may be obtained by contacting the Director of Licensing at Applied Biosystems, 850 Lincoln Center Dr., Foster City, California 94404 or Roche Molecular Systems, Inc., 1145 Atlantic Avenue, Alameda, California 94501.

• ABI (Applied Biosystems) Prism 7000, 7300, 7500, 7900HT, and StepOnePlusTM
are trademarks of Applera Corporation.
• Bio-Rad iCycler, IQ5, and CFX96 are trademarks of Bio-Rad.
• Qiagen Rotor-Gene is a trademark of Qiagen.
• LightCycler® 2.0 and 480 are trademarks of Roche.
• Cepheid Smartcycler II is a trademark of Cepheid.
• Stratagene MX3000PTM QPCR is a trademark of Stratagene.
• NucliSENS EasyMAG System is a trademark of Biomerieux.
• MagNa Pure LC® system is a trademark of Roche.
• QIASymphony® is a trademark of Qiagen.
• QIAamp® DNA Blood Mini Kit is a trademark of Qiagen.
• QIAamp® DNA Micro Kit is a trademark of Qiagen.
• QIAamp® DNA Stool Mini Kit is a trademark of Qiagen.
• QIAamp® DNA Mini Kit is a trademark of Qiagen.
• Maxwell 16 Blood DNA Purification Kit is a trademark of Promega.
• LC 480 Probe Master is a trademark of Roche.
• Optima DU Master Mix 5X DNA is a trademark of Diagenode.
• TaqMan Probe LC 2.0 is a trademark of Roche.
• QuantifastTM Multiplex PCR +R kit is a trademark of Qiagen.
• QuantifastTM Probe PCR +ROX Vial Kit is a trademark of Qiagen.
• Rotor-Gene Multiplex PCR Kit is a trademark of Qiagen.

Class Ea1: virus that may cause disease in animals and is characterized (to varying extents) by the following elements: limited geographical significance, low interspecies transmissibility, no vectors or carriers. Does not generally require any special containment measures. An effective prophylaxis and/or curative treatment is usually available.

DICD-XX-V02_EN_07_07_2016

Diagenode sa
Liège Science Park
Rue du Bois Saint-Jean, 3
4102 Seraing
BELGIUM

Tel. +32 4 364 20 50
info@diagenodediagnostics.com
www.diagenodediagnostics.com
